# Endorsing and Reinforcing Gender and Age Stereotypes: The Negative Effect on Self-Rated Leadership Potential for Women and Older Workers

**DOI:** 10.3389/fpsyg.2019.00688

**Published:** 2019-04-18

**Authors:** Fatima Tresh, Ben Steeden, Georgina Randsley de Moura, Ana C. Leite, Hannah J. Swift, Abigail Player

**Affiliations:** School of Psychology, University of Kent, Canterbury, United Kingdom

**Keywords:** gender, age, stereotypes, organizational culture, leadership potential

## Abstract

Previous research has examined the impact of stereotypes on outcomes such as career progression and hiring decisions. We present a novel approach to examine the role of stereotypes in predicting self-rated leadership potential across gender and age groups. This research sheds light on the impact of leadership-incongruent and detrimental stereotypes about one's gender and age, for women and older workers, on self-ratings of leadership potential. Across three studies (total *N* = 640), correlational and experimental evidence shows differential effects of stereotypes about women (vs. men) and older (vs. younger) people on self-ratings of their own leadership potential. Results suggest that both gender and age stereotypes affect older workers more than their younger counterparts (Study 1). Specifically, effects on self-rated leadership potential at the intersectional level show that endorsement of stereotypes has opposite effects on older women to younger men (Study 1). Furthermore, stereotyped workplace cultures impacted women's and older worker's perceptions of job fit (Studies 2 and 3), also extending to job appeal for older workers (Study 3). Results are discussed in terms of career implications for both women and older workers, with a particular focus on older women, whose intersecting identities are leadership stereotype-incongruent.

## Introduction

In order to maintain competitive advantage, organizations must identify and nurture people with high-potential to drive innovation (Salau et al., 2018), and ultimately succeed leaders (Stadler, 2011). To do this successfully, organizations should be able to identify those with the most leadership potential objectively, free from bias and subjectivity. However, observation of talent pools and leadership teams indicate that there are sociodemographic restrictions to identification of leadership potential. That is, younger men are disproportionately represented in leadership positions relative to their older and/or female counterparts (World Economic Forum, 2015; Business in the Community, 2016). We take a novel approach to the study of leadership potential by examining the psychological barriers that members of disadvantaged and stigmatized groups in the workplace may face in leadership attainment, because leadership stereotypes favor men and younger workers. Specifically, we investigate the relationship between stereotype endorsement and stereotype reinforcement on how men (vs. women) and younger (vs. older) workers judge their own leadership potential. We focus on gender and age as both have been found to impact assessments of others' leadership potential (Hirschfeld and Thomas, 2011; Tresh et al., 2018; Player et al., in press).

To address gender and age inequalities in the workplace, which are exacerbated by an aging workforce and increased representation of women in the workplace (Business in the Community, 2017; Catalyst, 2018), organizations need to diversify their leadership teams. Diversity in leadership teams has been linked with improved financial performance (McKinsey Company, 2015) and innovation (Bantel and Jackson, 1989). The challenges for disadvantaged gender and stigmatized age groups in talent identification cannot be due to objective differences in desired attributes, given that women and older workers perform objectively as well as their younger and male leadership counterparts (Eagly et al., 1995; Posthuma and Campion, 2009). A more plausible explanation is psychological biases against these groups in the form of subjective and unfavorable evaluations. Recent research has shown that gender is a boundary condition to the preference for potential (over past performance) in candidates for leadership positions (Player et al., in press). Specifically, we found that men are selected for leadership positions based on their future potential, whereas women are selected based on past performance (Player et al., in press). Furthermore, women are held to higher standards than men in order to be perceived as having leadership potential in the eyes of men who are making a promotion decision (Tresh et al., 2017).

The current studies examine the impact of (a) stereotype endorsement (Study 1) and (b) stereotype reinforcement (Studies 2 and 3), on how men vs. women and younger workers vs. older workers (e.g., Beck and Williams, 2016), rate their own potential to lead. Societal and workplace stereotypes have provided substantial evidence for biased evaluations against women (e.g., Eagly and Karau, 2002) and older workers (e.g., Abrams et al., 2016; Swift et al., 2017) with respect to their leadership suitability and performance. Our approach provides a useful perspective for understanding the negative effects stereotypes might have for attaining equal outcomes in terms of career choices and progression. The present research contributes to the growing body of literature challenging widely held prejudicial beliefs that workplace stereotypes of disadvantaged and stigmatized groups in the workplace are due to objective differences in traits and skills or individuals' sub-optimal career choices (e.g., Tam, 1997; Polavieja, 2012).

## Leadership Potential

“Leadership potential” is reserved by organizational evaluators for individuals who indicate likely effectiveness in future roles, usually with much broader responsibilities and at higher levels in the hierarchy (Silzer and Church, 2009). Early research on leadership potential has focused on the traits and skills which most accurately predict leadership success in the long-term (Hirschfeld et al., 2008; Silzer and Church, 2009; Dries and Pepermans, 2012). More recently, research has begun to consider the subjective nature of leadership potential (e.g., Peters and Haslam, 2018), and the challenges with identifying specific traits or skills (Tresh et al., 2018).

A small but consistent body of research has demonstrated a preference for leadership potential in leadership selection, such that candidates with leadership potential are preferred over candidates with more leadership achievement (e.g., Tormala et al., 2012; Sun et al., 2015). These findings seem to reflect organizational practice- a psychological preference for potential mirrors organizations' desire to identify leadership potential (Schwartz et al., 2017). It makes sense then to understand what affects evaluations of leadership potential, especially given that an understanding of potential impacts outcomes such as ambition and performance (Steffens et al., 2018).

Existing research into leadership potential has focused primarily on evaluator-candidate dyadic relationships, namely how an evaluator perceives the candidate's leadership potential (e.g., Heslin, 2009; Dries and Pepermans, 2012; Peters and Haslam, 2018). Our research presented here takes a new perspective by investigating *self-rated* leadership potential (i.e., the amount of leadership potential people attribute to themselves). Recent research conducted by Steffens et al. (2018) examined the consequences of receiving feedback about one's own leadership potential. Specifically, Steffens et al. (2018) showed that those who are told that they have low leadership potential show less ambition to become leaders and perform less well in subsequent tasks compared to those who are told that they have high leadership potential. As leadership ambition and performance are attributes used to identify leadership potential (Robinson et al., 2009; Silzer and Church, 2009; Dries and Pepermans, 2012), this can undoubtedly affect leadership attainment by increasing or reducing confidence in one's own leadership abilities. Little is known about the social-psychological antecedents of self-rated leadership potential and the extent to which this could be influenced by stereotypes about the social groups that individuals belong to. To address this gap in the literature, our research examines the effects of endorsing and reinforcing gender and age stereotypes on self-rated leadership potential. We expect that men and younger people will be advantaged by endorsing the leadership-congruent stereotypes about their own gender and age, and will rate themselves as having more leadership potential as a result of endorsing them. In contrast, we expect that women and older people will be disadvantaged by reinforced stereotyped workplace cultures to the extent that it impacts their job appeal, self-rated job fit, and self-rated leadership potential.

## Workplace Stereotypes and Leadership

There is no evidence that the underrepresentation of women in leadership roles is caused by women having insufficient skillsets to assume leadership positions (Gipson et al., 2017). Instead, research has highlighted the role of psychological biases, namely gender stereotypes, in perpetuating a gender bias in leadership (Hoyt, 2010). Much of this research is focused on how gender stereotypes lead to discriminatory practices against women, but less on how women themselves may be impacted by societal gender stereotypes.

The “think manager – think male” paradigm evidences the tendency to consolidate the representation of leadership with gender roles of men, because stereotypes of men and of leaders both reflect agency (e.g., independence, assertiveness, confidence). On the other hand, women are generally attributed “communal” traits typically not associated with leadership (e.g., kind, caring, cooperative) as described by role congruity theory (Eagly and Karau, 2002). Research has shown that these gender stereotypes influence children's behavior from an early age. For example, boys' perceptions of gender stereotypes are associated with their beliefs about the abilities of boys and girls, and predict self-rated competence (Kurtz-Costes et al., 2008). Moreover, girls' implicit gender stereotypes are predicted by their mothers' implicit gender stereotypes about children (Endendijk et al., 2013).

Studies in leadership selection have found that agentic and typically masculine attributes are preferred over communal and typically feminine attributes in recruitment decisions, advantaging male candidates (Sczesny, 2004). This effect is strengthened when the leadership role requires masculine-typed traits (Von Stockhausen et al., 2013). Furthermore, such biases can be held by women as well as men, with both women and men perceiving successful managers as holding attributes more associated with men than with women (Schein, 1973, 1975). Nonetheless, this finding highlights that women perceive successful managers as having stereotypically male attributes, but not necessarily that men are more suitable for leadership positions than women. Evidence shows that men can hold stronger gender biases than women (for a review see Atewologun et al., 2018), which echoes research in social psychology on high-status compared to low-status groups demonstrating stronger in-group biases (Bettencourt et al., 2001). Gender stereotypes give men a higher advantage in terms of leadership attainment, which might explain why men are more likely to endorse gender stereotypes than women (Mast, 2005).

Role congruity theory offers a model applicable to other sociodemographic groups as well as gender, such as age (Krings et al., 2011). Evidence has shown that older workers are equally, if not more productive and competent in the workplace (McCann and Giles, 2002; Posthuma and Campion, 2009). Nonetheless, research has highlighted that age stereotypes often result in bias toward hiring candidates who are perceived to possess stereotypically “younger” over “older” traits, and that this is replicated even when recruiting for higher-status roles (Abrams et al., 2016).

Age stereotypes are positioned along the warmth-competence dimensions, according to the stereotype content model (Fiske et al., 1999, 2002), with older people attributed greater “warmth” and less “competence.” Pervasive age stereotypes in the workplace suggest that older workers have lower performance, ability, technical competence, motivation, and productivity (Broadbridge, 2001; Cuddy and Fiske, 2002; Posthuma and Campion, 2009). Warmth stereotypes generally position older workers more positively, describing them as loyal and interpersonally skilled (Warr and Pennington, 1993). However, a preference for competence-related traits in leadership selection advantages younger candidates compared to older candidates with objectively equal resumés (Perry et al., 2017). In addition, older age stereotype characteristics, such as carefulness and politeness, can disadvantage older people in hiring decisions. Whereas, younger age stereotypes such as creativity, adaptability, flexibility, and greater willingness to learn new things can be preferred (Abrams et al., 2016). As for gender stereotypes, age stereotypes are likely to elicit the similar in-group bias for higher-status members (younger workers) than their marginalized counterparts (older workers) because age stereotypes about competence give younger workers a higher advantage in terms of leadership attainment (Finkelstein et al., 1995; Gordon and Arvey, 2004).

## Endorsing Ingroup Stereotypes

Although high-status groups are more likely to endorse advantageous group stereotypes (Finkelstein et al., 1995; Gordon and Arvey, 2004), theory and research highlight that gender and age stereotypes are internalized and can have a profound effect on people's self-definitions and behaviors. Theories such as gender schema theory (Bem, 1981) and the expectancy value model (Eccles et al., 1983) argue that gender stereotypes are culturally propagated, through mechanisms such as the socializing influence of parents, and internalized from childhood. For example, when female managers endorse gender stereotypes, they self-stereotype as strong in feminine skills and weak in masculine skills (Eiksson et al., 2017). This also applies to other contexts. For example, salient math-gender stereotypes about women's under-performance in math have been shown to reduce women's intentions to have a career in math, explained partly by internalized beliefs about their math competence (Song et al., 2017).

Stereotype embodiment theory (Levy, 2009) suggests that age stereotypes, which are learnt when people are young, can lead to similar self-stereotyping in older people. Culturally pervasive negative age stereotypes can become internalized throughout the life course and become increasingly salient and self-relevant as individuals age. Endorsement of negative age stereotypes that denote older people as physically and cognitively less capable than younger people has been found to impact negatively on older people's cognitive functioning, physical health (Wurm et al., 2007) and willingness to engage in physical activity (Emile et al., 2014). Furthermore, the Risks of Ageism Model (RAM) highlights how these stereotyping processes play out in employment contexts to disadvantage older workers (Swift et al., 2017). For instance, lack of perceived “fit” with the organization, lack of respect, and appreciation of older workers, are important factors that influence older workers intentions to exit the labor market. However, research has yet to show whether younger workers indeed perceive more leadership potential in themselves than older workers do, and whether this is explained by younger worker's greater likelihood of endorsing age stereotypes.

We address these gaps in the literature to examine the relationship between gender and age stereotype endorsement and self-rated leadership potential. We expect that greater endorsement of stereotypes by higher-status groups, in this case men and younger workers, will provide an explanation for higher self-rated leadership potential amongst these categories compared to women and older workers. Specifically:

***Hypothesis 1:*** *There will be a relationship between gender and self-rated leadership potential, such that men* (*vs. women*) *will have higher self-rated leadership potential*.

***Hypothesis 2:*** *We expect that the relationship between gender and self-rated leadership potential will be mediated by the endorsement of gender stereotypes*.

***Hypothesis 3:*** *There will be a relationship between age and self-rated leadership potential, such that younger people* (*vs. older people*) *will have higher self-rated leadership potential*.

***Hypothesis 4:*** *We expect that the relationship between age and self-rated leadership potential will be mediated by the endorsement of age stereotypes*.

## Reinforcing Ingroup Stereotypes

Evidence suggests that a context in which negative gender and age stereotypes are salient can have an immediate effect on women and older people's behavior. Experimental data shows that women are less willing to contribute ideas to a group when the area of expertise is incongruent with traditional gender roles or communal traits, regardless of other group members' behavior (Coffman, 2014). The salience of traditional gender roles and traits for women has also been shown to weaken some women's attitudes and increase their susceptibility to persuasion (Eaton et al., 2017). Similarly, when age stereotypes that denote older people as incompetent are made salient, older people approach tasks requiring high competence differently, becoming more cautious in eyewitness memory tasks (Thomas et al., 2018) and less confident in their driving ability despite consistent objective performance (Chapman et al., 2014). These findings are consistent with the stereotype embodiment theory and stereotype threat theory, such that increased anxiety about confirming negative in-group stereotypes in stereotyped domains leads to reduced performance (Steele, 1992, 1997). As such, contextual cues encourage age-stereotype-congruent beliefs and behaviors (Levy, 2009; Swift et al., 2017). However, research on the effects of negative age stereotypes have focused primarily on health and cognition, with less research exploring the effects on organizational outcomes.

Gender stereotypes are likely made salient during leadership evaluations because the evaluator will assess the *perceived* congruence between the individual (and their group) and the *perceived* requirements of the role (Eagly and Karau, 2002). However, there are other contextual factors that may affect the salience of gender stereotypes in leadership evaluations. One of these factors is organizational culture. The tendency for evaluators to assess the *perceived* congruence between the individual (and their group) and the *perceived* organizational culture (Sarris and Kirby, 2005) partly explains why women are more likely to be leaders in industries which value communal traits such as education and healthcare, and support functions such as human resources (e.g., Bowles et al., 2005; Gipson et al., 2017). This is also evidenced by a wider gender gap in leadership attainment in masculine-typed organizational cultures (Elesser and Lever, 2011); contexts that are more likely to elicit stereotype threat in women (Kray and Shirako, 2011). Research has shown that lack of fit for women affects access to networks and mentors, career capital necessary for leadership attainment (Simpson, 2000). Also, it is not yet known whether organizational cultures that reinforce gender stereotypes have spill-over effects on women's self-rated leadership potential. Previous research has found that women are particularly devalued when they occupy male-dominated leadership roles (Eagly et al., 1992). Therefore, we expect that female participants will perceive particularly low appeal, job fit and leadership potential in themselves when presented with masculine-typed organizational cultures. Given that stereotypes about men are congruent with leadership, we do not expect organizational culture (masculine or feminine) to have an effect on men's self-rated leadership potential.

***Hypothesis 5:*** *Gender and stereotype reinforcement* (*labeled organizational culture*) *will interact to predict job appeal, self-rated job fit, and self-rated leadership potential. Specifically, female participants will self-rate significantly less job appeal, job fit, and leadership potential in a masculine stereotyped culture than in a feminine stereotyped culture. Organizational culture will have no effect on male participants' job appeal, self-rated job fit, or self-rated leadership potential*.

As with gender, contextual factors likely affect the salience of age stereotypes in leadership evaluations. An evaluator will likely assess the *perceived* congruence between the older candidate (and their group) with the *perceived* requirements of the role, as well as their *perceived* fit with the organizational culture (Swift et al., 2017). This explains why organizational culture influences preferences for younger (vs. older) candidates. For example, older candidates are preferred in times of stability (Spisak et al., 2014) and in traditional, stable companies (Diekman and Hirnisey, 2007). Perceptions of job fit and job appeal also predict older people's intentions to exit the workforce (Swift et al., 2017). As such, we would expect organizational culture to have the same impact on job appeal and self-rated job fit, as demonstrated in the literature, for older participants as we do for female participants. We would expect this to extend to self-rated leadership potential.

***Hypothesis 6:*** *Age and stereotype reinforcement* (*manipulated as organizational culture*) *will interact to predict job appeal, self-rated job fit, and self-rated leadership potential. Specifically, older people will self-rate significantly less job appeal, job fit, and leadership potential in a younger stereotyped culture than in an older stereotyped culture. Organizational culture will have no effect on younger people's job appeal, self-rated job fit or self-rated leadership potential*.

## Overview of Research

This research applies a well-established literature on stereotypes to the understanding of leadership potential and how it is subjectively perceived by individuals.

In Study 1, we explore the relationship between stereotype endorsement and self-rated leadership potential. We then examine the effects of stereotype reinforcement (i.e., in the form of organizational culture) on self-rated job appeal, job fit, and leadership potential in Studies 2 and 3. All studies were pre-registered via the Open Science Framework (https://osf.io/83rf2/, https://osf.io/rqhpm/, https://osf.io/j6rm5/). All studies have ethical approval, following the authors' institutional psychology ethics process.

## Study One

In Study 1, we test our Hypotheses 1–2 (gender) and 3–4 (age), and analyze the mediation effects of stereotype endorsement on individuals' self-rated leadership potential.

### Method

#### Participants and Design

Participants were recruited via the online crowdsourcing platform Prolific. We recruited 276 participants initially; 19 participants either failed the attention check, provided identifiable information or timed-out after 20 min so their data was not included in the analysis. Total participant numbers comprised 128 men, 124 women, and 2 participants who did not identify as either male or female^1^. Participants were recruited in one of two age categories: 126 participants were in the 18–30 category (*M* = 25.54, *SD* = 3.16) and 126 participants were in the age 50 and older category (*M* = 55.80, *SD* = 4.98)^2^. The total number of participants included in the analysis was 252. All participants were in full- or part-time employment in the UK. Participants received a payment of £0.50 and the average completion time was 354.77 s (*SD* = 139.90).

Study 1 adopted a correlational design. We measured the relationships between participant gender, endorsement of agentic and communal (gender) stereotypes, and self-rated leadership potential. We also measured the relationships between participant age, endorsement of competence and warmth (age) stereotypes and self-rated leadership potential.

### Procedure and Materials

Participants were invited to take part in an online survey on Qualtrics (survey software) to understand self-perceptions. They were informed that data would be treated confidentially, would be anonymized for publication, and that participation was voluntary and could be withdrawn at any time. Email contact details for two of the researchers were also supplied, and participants gave their informed consent by clicking to take part in the study. Participants then completed the measures as defined below. Participants were finally presented with a full debrief of the study, and reminded of the researchers' contact details.

### Measures

All questions were scored on a seven-point scale (1 = *very much disagree*, 7 = *very much agree*).

#### Gender Stereotype Endorsement

Endorsement of gender stereotypes was measured using 14 items asking participants “*please indicate the extent to which you agree with the following statements… Female workers are more communal/ supportive/ competitive/ kinship-oriented/ warmer/ kind/ assertive/ nicer/ stronger/ self-sufficient/ independent/ cooperative/ capable/ confident than male workers.”* Gender stereotype descriptors were sourced from the existing literature (e.g., Eagly and Karau, 2002; Ridgeway, 2011). Items indicating agentic traits were reverse-coded, these included: competitive, assertive, stronger, self-sufficient, independent, and confident.

A mean score was used as the index of endorsement of seven agentic stereotypes (α = 0.89) and seven communal stereotypes (α = 0.89), with higher scores indicating greater endorsement of gender stereotypes. For example, higher scores on agentic stereotypes indicated attitudes that men are more agentic than women. Higher scores on communal stereotypes indicated attitudes that women are more communal than men.

#### Age Stereotype Endorsement

Endorsement of age stereotypes was measured using 20 items adapted from the “Work-related age-based stereotypes scale” (Marcus et al., 2016) asking participants “*please indicate the extent to which you agree with the following statements… Older workers are more intellectually competent/ achieve more/ physically capable/ better performers/ productive/ skilled/ perform worse/ suitable for training/ possess greater potential/ learn faster/ more flexible/ able to learn new things/ waste time training/ waste time and money training/ warm-hearted/ warmer personalities/ likable/ cold/ kind/ friendly than younger workers.”* Items indicating competent or adaptable traits were reverse-coded, as were “negative warm” traits, these included: intellectually competent, achieve more, physically capable, better performers, productive, skilled, suitable for training, possess greater potential, learn faster, more flexible able to learn new things and cold.

The scale measured three dimensions: competence (*N* = 7, α = 0.73), warmth (*N* = 6, α = 0.86), and adaptability (*N* = 7, α = 0.68). Given that no hypotheses were made about adaptability stereotypes and given that this scale had low reliability we did not include this subscale in the analyses reported below^3^. Competence had a low reliability and therefore the scale was reduced to 6 items, omitting the item on physical capability.

A final mean score was used as the index of endorsement of competence stereotypes (α = 0.81) and warmth stereotypes (α = 0.86), with higher scores indicating greater endorsement of age stereotypes. For example, a high score on competence stereotypes reflected attitudes that younger people are more competent than older people. A high score on warmth stereotypes indicated attitudes that older people are warmer than younger people.

#### Self-Rated Leadership Potential

Ratings of one's own leadership potential was measured using 7 items (three items adapted from Tresh et al., 2018, and four items adapted from Mueller et al., 2010) asking participants “*please indicate the extent to which you think you personally have the following… leadership potential/ the potential to become a successful leader/ the capability to be a leader/ the potential to become an effective leader/ the potential to develop leadership skills/ the potential to advance to a leadership position/ the potential to be a leader who is a role model for my co-workers.”* A mean score was used as the index of leadership potential (α = 0.97), with higher scores indicating higher self-rated leadership potential^4^.

### Results

Means and standard deviations for all measures and the bivariate correlations between the variables are reported in Table 1. We report the analyses by gender and then by age.

**Table 1:** Study 1: means, standard deviations and correlation matrix for specified variables.

	**M(SD)**	**1**	**2**	**3**	**4**	**5**	**6**	**7**
1. Gender			−0.02	−0.23^**^	0.06	0.03	−0.02	−0.03
2. Age				−0.05	−0.00	−0.29^**^	0.27^**^	−0.13^*^
3. Agentic stereotypes	4.38 (0.87)				−0.57^**^	0.33^**^	−0.41^**^	−0.12
4. Communal stereotypes	4.18 (0.89)					−0.40^**^	0.42^**^	0.08
5. Competence stereotypes	3.94 (0.86)						−0.70^**^	−0.01
6. Warmth stereotypes	4.02 (0.90)							−0.04
7. Leadership potential	5.07 (1.23)							

** *Correlation significant at the 0.01 level (2-tailed)*.

* *Correlation significant at the 0.05 level (2-tailed)*.

#### Gender

##### Hypothesis testing

We ran Pearson's bivariate correlations to establish the relationships between gender and endorsement of agentic stereotypes, endorsement of communal stereotypes and self-rated leadership potential. Failing to support Hypothesis 1, gender and self-rated leadership potential were not significantly associated; *r*_(250)_ = −0.03, *p* = 0.63. In partial support of hypothesis 2, there was a significant relationship between gender and endorsement of agentic stereotypes, such that men were more likely to endorse agentic stereotypes *r*_(250)_ = −0.23, *p* < 0.001. There was no relationship between gender and endorsement of communal stereotypes; *r*_(250)_ = 0.06, *p* = 0.34.

To test whether there was an indirect effect between gender and self-rated leadership potential via endorsement of gender stereotypes (Hypothesis 2), we used PROCESS macro (Model 4; see Hayes, 2013 with 5,000 bootstraps) with gender as the predictor (0 = men, 1 = women), endorsement of agentic and communal stereotypes as mediators (agentic in model 1, communal in model 2), and self-rated leadership potential as the outcome.

Results showed that gender was a significant predictor of endorsement of agentic stereotypes, such that men were more likely to endorse agentic stereotypes than women (*b* = −0.39, *SE* = 0.11, *t* = −3.70, *p* < 0.001, 95% CI −0.60, −0.18). Endorsement of agentic stereotypes was a significant, negative predictor of self-rated leadership potential; (*b* = −0.19, *SE* = 0.09, *t* = −2.11, *p* = 0.04, 95% CI −0.37, −0.01). The direct (*b* = −0.15, *SE* = 0.16, *t* = −0.95, *p* = 0.34, 95% CI −0.46, 0.16) and total effects were non-significant (*b* = −0.07, *SE* = 0.15, *t* = −0.48, *p* = 0.63, 95% CI −0.38, 0.23). The indirect effect was non-significant (Indirect effect: *b* = 0.08, *SE* = 0.05, 95% CI −0.02, 0.20).

Gender was not a significant predictor of endorsement of communal stereotypes (*b* = 0.11, *SE* = 0.11, *t* = 0.96, *p* = 0.34, 95% CI −0.11, 0.33), and endorsement of communal stereotypes was not a significant predictor of self-rated leadership potential (*b* = 0.12, *SE* = 0.09, *t* = 1.34, *p* = 0.18. 95% CI −0.05, 0.29). The direct (*b* = −0.09, *SE* = 0.15, *t* = −0.56, *p* = 0.58, 95% CI −0.39, 0.22) and total effects were non-significant (*b* = −0.07, *SE* = 0.15, *t* = −0.48, *p* = 0.63, 95% CI −0.38, 0.23). The indirect effect was non-significant (*b* = 0.01, *SE* = 0.02, 95% CI −0.02, 0.07).

##### Moderation analyses

Although we found that men were more likely to endorse agentic stereotypes than women, this did not relate to self-rated leadership potential possibly because there was no difference between self-rated leadership potential for men and women. However, it is possible that for women who do endorse gender stereotypes, there is a negative relationship with self-rated leadership potential that does not occur for men. To test this possibility, we conducted exploratory moderation analyses to test the interactive effects of endorsement of gender stereotypes and gender on self-rated leadership potential (using model 1 in PROCESS, Hayes, 2013). We introduced gender stereotypes as predictors (agentic in model 1, communal in model 2), participant gender as a moderator, and perceptions of self-leadership potential as the outcome. Results were non-significant (see Table 2).

**Table 2:** Study 1: exploratory moderated regression analysis for gender stereotypes.

**Items**	**B**	***SE* B**	***t***	***p***	**LCI**	**UCI**
**AGENTIC**						
Agentic stereotypes	0.21	0.28	0.74	0.46	−0.35	0.76
Gender	1.05	0.81	1.29	0.20	−0.55	2.65
Agentic stereotypes x Gender	−0.28	0.18	−1.50	0.13	−0.64	0.09
**COMMUNAL**						
Communal stereotypes	−0.08	0.27	−0.30	0.77	−0.61	0.45
Gender	−0.65	0.75	−0.87	0.39	−2.13	0.82
Communal stereotypes x Gender	0.13	0.17	0.77	0.44	−0.21	0.48

### Age

#### Hypothesis testing

To test Hypothesis 3, we ran Pearson's bivariate correlations to establish relationships between age and endorsement of competence stereotypes, endorsement of warmth stereotypes, and self-rated leadership potential. In support of Hypothesis 3, there was a significant relationship between age and self-rated leadership potential; *r*_(250)_ = −0.13, *p* = 0.04. Younger workers rated more leadership potential in themselves and older workers rated less leadership potential in themselves. There was a significant relationship between age and endorsement of competence stereotypes; *r*_(250)_ = −0.29, *p* < 0.001, and age and endorsement of warmth stereotypes; *r*_(250)_ = 0.27, *p* < 0.001. In partial support of Hypothesis 4, younger people were more likely to endorse competence stereotypes than older people, and contrary to Hypothesis 4, they were less likely to endorse warmth stereotypes than older people.

To test whether there was an indirect effect between age and self-rated leadership potential via age stereotypes (Hypothesis 4), we used PROCESS macro (Model 4; see Hayes, 2013 with 5,000 bootstraps) with age as the predictor (0 = younger people, 1 = older people), endorsement of competence and warmth stereotypes as mediators (competence in model 1, warmth in model 2) and self-rated leadership potential as the outcome.

Results showed that age was a significant predictor of endorsement of competence stereotypes, such that younger people were more likely to endorse competence stereotypes (*b* = −0.50, *SE* = 0.10, *t* = −4.81, *p* < 0.001, 95% CI −0.71, −0.30). Endorsement of competence stereotypes was not a predictor of self-rated leadership potential (*b* = −0.07, *SE* = 0.09, *t* = −0.78, *p* = 0.44, 95% CI −0.26, −0.11). The direct (*b* = −0.36, *SE* = 0.16, *t* = −2.25, *p* = 0.03, 95% CI −0.68, −0.05) and total effects were significant (*b* = −0.33, *SE* = 0.15, *t* = −2.12, *p* = 0.03, 95% CI −0.63, 0.02). The indirect effect was non-significant (*b* = 0.04, *SE* = 0.06, 95% CI −0.07, 0.16).

Results showed that age was a significant predictor of endorsement of warmth stereotypes, such that younger people were less likely to endorse warmth stereotypes (*b* = 0.49, *SE* = 0.11, *t* = 4.49, *p* < 0.001, 95% CI 0.28, 0.71), but endorsement of warmth stereotypes was not a predictor of self-rated leadership potential (*b* = −0.01, *SE* = 0.09, *t* = −0.09, *p* = 0.93, 95% CI −0.18, 0.17). The direct (*b* = −0.32, *SE* = 0.16, *t* = −2.01, *p* = 0.05, 95% CI −0.64, −0.01) and total effects were significant (*b* = −0.33, *SE* = 0.15, *t* = −2.12, *p* = 0.03, 95% CI −0.63, −0.02). The indirect effect was non-significant (*b* = −0.004, *SE* = 0.06, 95% CI −0.12, 0.12).

#### Moderation analyses

We found support for Hypothesis 3, younger people were associated with higher self-rated leadership potential. Furthermore, we found partial support for Hypothesis 4 because younger people were more likely to endorse competence stereotypes than older people. However, this did not relate to self-rated leadership potential. It is possible that for older workers who do endorse age stereotypes, there is a negative relationship with self-rated leadership potential that does not occur for younger workers. We conducted exploratory moderation analyses to test the interactive effects of endorsement of age stereotypes and age on self-rated leadership potential (using model 1 in PROCESS, Hayes, 2013). We introduced age stereotypes as predictors (competence in model 1, warmth in model 2), participant age as a moderator, and self-rated leadership potential as the outcome. Results were non-significant (see Table 3).

**Table 3:** Study 1: exploratory moderated regression analysis for age stereotypes.

**Items**	**B**	***SE* B**	***t***	***p***	**LCI**	**UCI**
**COMPETENCE**						
Competence stereotypes	0.18	0.29	0.62	0.54	−0.39	0.74
Age	0.31	0.75	0.42	0.68	−1.16	1.79
Competence stereotypes x Gender	−0.17	0.19	−0.92	0.36	−0.54	0.20
**WARMTH**						
Warmth stereotypes	0.11	0.27	0.42	0.67	−0.42	0.65
Age	0.03	0.75	0.04	0.96	−1.45	1.52
Warmth stereotypes x Gender	−0.09	0.18	−0.48	0.63	−0.45	0.27

### Gender and Age

Analyzing gender and age separately we found little evidence of a relationship between endorsing in-group stereotypes and reduced self-rated leadership potential for women and older people, respectively. What we did not examine is how the intersecting identities of these groups may respond to stereotypes with regards to either their age or gender. The literature on discrimination toward older women indicates that a combined identity of being leadership-incongruent in terms of both gender and age may have more pronounced effects than being leadership-incongruent based on a single identity (Duncan and Loretto, 2004). This is echoed in the healthcare context, where internalized negative stereotypes have a cumulative burden on older women, reducing health care seeking behaviors (Chrisler et al., 2016). It is possible that the burden of negative stereotypes that relate to older women's gender and age have a similar effect on their self-rated potential to lead.

We conducted exploratory moderation analyses to test the main and interactive effects of gender and age, with endorsement of gender and age stereotypes, on self-rated leadership potential at the intersectional level of identity (using model 3 in PROCESS, Hayes, 2013). In total, we tested four models: agentic stereotypes (model 1), communal stereotypes (model 2), competence stereotypes (model 3), and warmth stereotypes (model 4). Results of the three-way interactions are reported in text because we are particularly interested in the intersection of age and gender, all other effects are reported in full in Table 4.

**Table 4:** Study 1: three-way interaction between endorsement of stereotypes, participant gender and participant age on self-rated leadership potential.

**Items**			**B**	***SE***	***t***	***R*^**2**^**	**Δ*R*^**2**^**	***F***	**df**	***p***	**LCI**	**UCI**
**Agentic**						0.23	0.05	2.00	7,244	0.06		
Agentic stereotypes			−0.99	0.88	−1.13					0.26	−2.71	0.74
Gender			−2.86	2.57	−1.11					0.27	−7.91	2.20
Age			−3.69	2.58	−1.43					0.15	−8.77	1.39
Agentic stereotypes x Gender			0.61	0.58	1.06					0.29	−0.52	1.75
Agentic stereotypes x Age			0.76	0.57	1.34					0.18	−0.36	1.87
Gender x Age			2.48	1.63	1.52					0.13	−0.73	5.69
Agentic stereotypes x Gender x Age			−0.57	0.37	−1.54					0.12	−1.29	0.16
							0.01	2.37	1,244	0.12		
**Communal**						0.24	0.06	2.19	7,244	0.04		
Communal stereotypes			2.11	0.81	2.62					0.01	0.53	3.70
Gender			5.02	2.27	2.21					0.03	0.55	9.49
Age			6.20	2.33	2.66					0.01	1.60	10.79
Communal stereotypes x Gender			−1.22	0.53	−2.32					0.02	−2.27	−0.18
	Younger workers							1.37	1,244	0.24		
	Older workers							6.71	1,244	0.01		
Communal stereotypes x Age			−1.57	0.55	−2.85					0.005	−2.65	−0.49
	Men							6.25	1,244	0.01		
	Women							1.90	1,244	0.17		
Gender x Age			−4.00	1.50	−2.66					0.01	−6.95	−1.04
Communal stereotypes x Gender x Age						0.06	0.03	7.44	1,244	0.01		
			0.96	0.35	2.73					0.01	0.27	1.65
		Younger men	0.28	0.15	1.92					0.06	−0.01	0.57
		Younger women	0.01	0.18	0.08					0.94	−0.33	0.36
		Older men	−0.33	0.20	−1.69					0.09	−0.71	0.06
		Older women	0.36	0.18	1.99					0.05	0.003	0.72
**Competence**						0.22	0.05	1.83	7,244	0.08		
Competence stereotypes			−1.53	0.87	−1.76					0.08	−3.25	0.19
Age			−4.72	2.30	−2.06					0.04	−9.24	−0.20
Gender			−4.77	2.46	−1.94					0.05	−9.61	0.07
Competence stereotypes x Age			1.15	0.57	2.00					0.05	0.02	2.28
	Men							0.88	1,244	0.35		
	Women							5.73	1,244	0.02		
Competence stereotypes x Gender			1.20	0.59	2.04					0.04	0.04	2.36
	Younger workers							1.21	1,244	0.27		
	Older workers							5.24	1,244	0.02		
Age x Gender			3.49	1.52	2.30					0.02	0.50	6.49
Competence stereotypes x Age x Gender			−0.91	0.38	−2.42					0.02	−1.66	−0.17
						0.05	0.02	5.85	1,244	0.02		
		Younger men	−0.10	0.16	−0.62					0.54	−0.41	0.22
		Younger women	0.19	0.20	0.91					0.36	−0.22	0.59
		Older men	0.14	0.19	0.71					0.48	−0.24	0.51
		Older women	−0.49	0.20	−2.50					0.01	−0.88	−0.10
**Warmth**						0.24	0.06	2.15	7,244	0.04		
Warmth stereotypes			2.42	0.83	2.93					0.004	0.79	4.05
Age			6.91	2.35	2.95					0.004	2.29	11.53
Gender			6.21	2.15	2.88					0.004	1.97	10.45
Warmth stereotypes x Age			−1.76	0.56	−3.14					0.002	−2.87	−0.66
	Men							6.46	1,244	0.01		
	Women							3.68	1,244	0.06		
Warmth stereotypes x Gender			−1.57	0.54	−2.92					0.004	−2.63	−0.51
	Younger workers							3.61	1,244	0.06		
	Older workers							6.29	1,244	0.01		
Age x Gender			−4.63	1.50	−3.10					0.002	−7.57	−1.68
Warmth stereotypes x Age x Gender			1.13	0.36	3.14					0.002	0.42	1.84
						0.06	0.04	9.88	1,244	0.002		
		Younger men	0.22	0.15	1.44					0.15	−0.08	0.52
		Younger women	−0.22	0.17	−1.26					0.21	−0.56	0.12
		Older men	−0.41	0.20	−2.10					0.04	−0.80	−0.03
		Older women	0.28	0.20	1.45					0.15	−0.10	0.67
		Younger men	−0.10	0.16	−0.62					0.54	−0.41	0.22
		Younger women	0.19	0.20	0.91					0.36	−0.22	0.59
		Older men	0.14	0.19	0.71					0.48	−0.24	0.51
		Older women	−0.49	0.20	−2.50					0.01	−0.88	−0.10
**Warmth**						0.24	0.06	2.15	7,244	0.04		
Warmth stereotypes			2.42	0.83	2.93					0.004	0.79	4.05
Age			6.91	2.35	2.95					0.004	2.29	11.53
Gender			6.21	2.15	2.88					0.004	1.97	10.45
Warmth stereotypes x Age			−1.76	0.56	−3.14					0.002	−2.87	−0.66
	Men							6.46	1,244	0.01		
	Women							3.68	1,244	0.06		
Warmth stereotypes x Gender			−1.57	0.54	−2.92					0.004	−2.63	−0.51
	Younger workers							3.61	1,244	0.06		
	Older workers							6.29	1,244	0.01		
Age x Gender			−4.63	1.50	−3.10					0.002	−7.57	−1.68
Warmth stereotypes x Age x Gender			1.13	0.36	3.14					0.002	0.42	1.84
						0.06	0.04	9.88	1,244	0.002		
		Younger men	0.22	0.15	1.44					0.15	−0.08	0.52
		Younger women	−0.22	0.17	−1.26					0.21	−0.56	0.12
		Older men	−0.41	0.20	−2.10					0.04	−0.80	−0.03
		Older women	0.28	0.20	1.45					0.15	−0.10	0.67

### Agentic Stereotypes

We introduced endorsement of agentic stereotypes as a predictor, and participant gender and participant age as moderators, with self-rated leadership potential as the outcome.

Results showed no main effects of endorsement of agentic stereotypes, participant gender, or participant age. There were no interaction effects.

### Communal Stereotypes

We introduced endorsement of communal stereotypes as a predictor, and participant gender and participant age as moderators, with self-rated leadership potential as the outcome.

Results showed significant main effects of endorsement of communal stereotypes, participant gender, and participant age on self-rated leadership potential. All two-way interaction effects were significant.

Results showed a significant interaction between endorsement of communal stereotypes (that women are more communal than men), participant gender and participant age (*b* = 0.96, *SE* = 0.35, *t* = 2.73, *p* = 0.01, 95% CI 0.27, 1.65). Conditional effects showed that endorsement of communal stereotypes had differential effects across age groups for men, *F*_(1, 244)_ = 6.25, *p* = 0.01, but not women, *F*_(1, 244)_ = 1.90, *p* = 0.17. Endorsement of communal stereotypes was marginally associated with higher self-rated leadership potential for younger men (*b* = 0.28, *SE* = 0.15, *t* = 1.92, *p* = 0.06, 95% CI −0.01, 0.57) but not older men (*b* = −0.33, *SE* = 0.20, *t* = −1.69, *p* = 0.09, 95% CI −0.71, 0.06). Conditional effects showed that endorsement of communal stereotypes had differential effects across gender for older workers; *F*_(1, 244)_ = 6.71, *p* = 0.01, but not younger workers [*F*_(1, 244)_ = 1.37, *p* = 0.24]. Endorsement of communal stereotypes was associated with higher self-rated leadership potential for older women (*b* = 0.36, *SE* = 0.18, *t* = 1.99, *p* = 0.05, 95% CI 0.003, 0.72) but not older men (*b* = −0.33, *SE* = 0.20, *t* = −1.69, *p* = 0.09, 95% CI −0.71, 0.06).

### Competence Stereotypes

We introduced endorsement of competence stereotypes as a predictor, and participant age and participant gender as moderators, with self-rated leadership potential as the outcome.

Results showed a marginally-significant main effect of endorsement of competence stereotypes and significant main effects of participant gender and participant age on self-rated leadership potential. All two-way interactions were significant.

Results showed a significant interaction between endorsement of competence stereotypes (that younger people are more competent than older people), participant age and participant gender (*b* = −0.91, *SE* = 0.38, *t* = −2.42, *p* = 0.02, 95% CI −1.66, −0.17). Conditional effects showed that endorsement of competence stereotypes had differential effects across gender for older workers, *F*_(1, 244)_ = 5.24, *p* = 0.02, but not younger workers *F*_(1, 244)_ = 1.21, *p* = 0.27. Endorsement of competence stereotypes was associated with lowered self-rated leadership potential in older women (*b* = −0.49, *SE* = 0.20, *t* = −2.50, *p* = 0.01, 95% CI −0.88, −0.10) but not older men (*b* = 0.14, *SE* = 0.19, *t* = 0.71, *p* = 0.48, 95% CI −0.24, 0.51). Conditional effects showed that endorsement of competence stereotypes had differential effects across age groups for women, *F*_(1, 244)_ = 5.73, *p* = 0.02, but not men, *F*_(1, 244)_ = 0.88, *p* = 0.35. Endorsement of competence stereotypes was associated with lowered self-rated leadership potential in older women (*b* = −0.49, *SE* = 0.20, *t* = −2.50, *p* = 0.01, 95% CI −0.88, −0.10), but not younger women (*b* = 0.19, *SE* = 0.20, *t* = 0.91, *p* = 0.36, 95% CI −0.22, 0.59).

### Warmth Stereotypes

We introduced endorsement of warmth stereotypes as a predictor, and participant age and participant gender as moderators, with self-rated leadership potential as the outcome.

Results showed significant main effects of endorsement of warmth stereotypes, participant gender, and participant age on self-perceived leadership potential. All two-way interaction effects were significant.

Results showed a significant interaction between endorsement of warmth stereotypes (that older people are warmer than younger people), participant age and participant gender (*b* = 1.13, *SE* = 0.36, *t* = 3.14, *p* = 0.002, 95% CI 0.42, 1.84). Conditional effects showed that endorsement of warmth stereotypes had differential effects across gender for older workers; *F*_(1, 244)_ = 6.29, *p* = 0.01, and marginally-significant effects for younger workers; *F*_(1, 244)_ = 3.61, *p* = 0.06. Endorsement of warmth stereotypes was associated with lower self-rated leadership potential for older men (*b* = −0.41, *SE* = 0.20, *t* = −2.10, *p* = 0.04, 95% CI −0.80, −0.03) but not older women (*b* = 0.28, *SE* = 0.20, *t* = 1.45, *p* = 0.15, 95% CI −0.10, 0.67). There were no effects for younger men (*b* = 0.22, *SE* = 0.15, *t* = 1.44, *p* = 0.15, 95% CI −0.08, 0.52) or younger women (*b* = −0.22, *SE* = 0.17, *t* = −1.26, *p* = 0.21, 95% CI −0.56, 0.12). Conditional effects showed that endorsement of warmth stereotypes had differential effects across age groups for men; *F*_(1, 244)_ = 6.46, *p* = 0.01, and marginally-significant effects for women; *F*_(1, 244)_ = 3.68, *p* = 0.06. Endorsement of warmth stereotypes was associated with lowered self-rated leadership potential for older men (*b* = −0.41, *SE* = 0.20, *t* = −2.10, *p* = 0.04, 95% CI −0.80, −0.03), but not younger men (*b* = 0.22, *SE* = 0.15, *t* = 1.44, *p* = 0.15, 95% CI −0.08, 0.52). There were no effects for younger women (*b* = −0.22, *SE* = 0.17, *t* = −1.26, *p* = 0.21, 95% CI −0.56, 0.12) or older women (*b* = 0.28, *SE* = 0.20, *t* = 1.45, *p* = 0.15, 95% CI −0.10, 0.67).

## Discussion

The results of Study 1 demonstrate the effects of leadership-incongruent stereotypes across gender and age groups on self-rated leadership potential. Across the age stereotypes, the effects were negative for older people but this was dependent on gender. Specifically, endorsing stereotypes about older people's warmth was associated with reduced self-rated leadership potential for older men but not older women. Furthermore, endorsing stereotypes about older people's competence was associated with reduced self-rated leadership potential for older women but not older men. Nonetheless, older women had higher self-rated leadership potential the more they endorsed communal stereotypes about women, something that younger women did not benefit from. Interestingly, endorsing stereotypes about women's communality was associated with high self-rated leadership potential in younger men. None of the stereotypes related to self-rated leadership potential for younger women. Overall, the results indicate that endorsing stereotypes about both gender and age have some negative impact on older people but not younger people.

We found that gender was not directly related to self-rated leadership potential. Although this failed to support Hypothesis 1, results revealed that men are more likely to endorse agency-based gender stereotypes, partially supporting Hypothesis 2, but this did not translate to higher self-rated leadership potential. In support of Hypothesis 3, we found that age was directly related to self-rated leadership potential. Although younger people were more likely to endorse competency-based age stereotypes, we did not find a mediation effect, failing to support Hypothesis 4.

Our exploratory analyses shed light on intersectionality issues. There was no negative interaction of gender or age stereotypes for neither younger men nor younger women. For younger men this would be expected given that gender stereotypes are leadership-congruent based on both their gender and age. However, we would expect gender stereotypes to interact for self-rated leadership potential for younger women to some degree because their gender (but not age) identity is leadership-incongruent. Perhaps in this study the salience of their age counteracted the negative effects of gender stereotypes, something to investigate in future research.

Age interacted with both age stereotypes and gender stereotypes. Endorsing age stereotypes around competency was detrimental for older women in terms of self-rated leadership potential, compared to their male and younger counterparts. Endorsement of warmth stereotypes had a potentially negative relationship with self-rated leadership potential for older men. Perhaps for older men, the warmth associated with aging becomes more salient than the agency associated with their gender. This was the opposite for older women, whose self-rated leadership potential increased when they endorsed communal traits about women. Interestingly, agency-based gender stereotypes had no interactive effects. Perhaps the nuances in the intersectional identities of these groups warrants further exploration. For example, intersectional identities become embedded within one another, interacting to form one unique identity through which inequality is experienced (Harnois, 2014; Martin et al., 2018).

Our findings could be explained by a generational difference between our participant groups. That is, older people may be more sensitive to all societal stereotypes than younger people. Our findings may also reflect the gendered nature of age stereotypes which may explain why age stereotypes were related to self-rated leadership potential differently for older men and older women. Martin et al. (2018) explain the gendered expectations of men and women as they age, showing that older men are expected to “lose” their agency. We found older men who endorse this, indicated by endorsement of older people's warmth, have lowered self-rated leadership potential.

Although we did not find a relationship between gender and self-rated leadership potential, we found this relationship for age. Perhaps the salience of societal gender roles is diminishing, or younger women better “manage” pervasive workplace stereotypes than their older counterparts. However, typical organizational cultures are often stereotypically masculine and younger with respect to the organizational norms, attitudes and behaviors endorsed within the workplace. As women and older people make decisions about job opportunities, these stereotyped cultures are likely to become more salient (Cochran et al., 2013; Kulik et al., 2016). We examine the role of stereotyped organizational cultures for women and older people's self-rated leadership potential in Studies 2 and 3.

## Study Two

In Study 2 we experimentally test Hypothesis 5 to examine whether the salience of a “masculine” organizational culture, compared to a “feminine” organizational culture, will reduce women's job appeal, self-rated job fit, and self-rated leadership potential.

### Method

#### Participants and Design

We recruited 228 participants through Prolific crowdsourcing platform. We removed 29 participants for the same reasons as outlined in Study 1, no participants were removed based on their responses to manipulation checks, reaching a final sample of 199 participants (94 men, 105 women, 18–67 years; *M* = 36.00, *SD* = 8.52). All participants were in full- or part-time employment. Participants received a payment of £0.95 and the average completion time was 342.00 (*SD* = 145.65).

The study adopted a 2 Participant gender (men vs. women) x 2 Workplace culture (agentic vs. communal) quasi-experimental mixed design. Participant gender was a between-participants variable, whereas workplace culture was a within-participants variable. Dependent variables measured job appeal, job fit and self-rated leadership potential.

### Procedure and Materials

Participants were invited to take part in an online survey on Qualtrics exploring people's job choices. They were provided with the same consent information as in study 1 and gave informed consent by clicking to continue. Participants were presented randomly with the masculine or feminine workplace culture condition first or second. In each condition, participants initially viewed a fictional online job advert for a leader in a UK-based company. The job adverts for both conditions began with the phrase “We are recruiting new leaders in the UK!” and were identically presented and worded, except for the name of the company to ensure meaningful comparison (“The Smith Group” or “The Jones Group”) and descriptors that were linked with either masculine or feminine workplace stereotypes. The descriptors used in the masculine workplace condition were: *independent, competitive, confident, assertive*, and *providing autonomy*; those used for the feminine workplace condition were: *cooperative, warm, supportive, connecting with people*, and *providing communality*. Descriptors were sourced from the existing literature (e.g., Eagly and Karau, 2002). No other information on the type of employer, such as size or industry, was included.

Participants completed a manipulation check and dependent measures after each advert before reviewing both adverts again and answering dependent measure choice-questions. Participants completed demographic questions on age, gender and ethnic origin and were finally presented with a full debrief.

### Measures

Questions were scored on a seven-point scale (1 = *very much disagree*, 7 = *very much agree*), with the exception of choice questions.

#### Manipulation Checks

To measure the extent to which participants perceived the organization to be masculine or feminine, participants indicated their agreement with two items: “*Think about this job advert and please indicate the extent to which you agree with the following… women would enjoy this job/ men would enjoy this job.”*

#### Job Appeal

Job appeal was measured using 5 items (adapted from Gaucher et al., 2011) asking participants “*please indicate to what extent you agree or disagree with the following statements… I think I could enjoy this job/ this is not a job I would want/ this company would be a good employer/ this job looks interesting/ this company seems like a great place to work.”* A mean score was used as the index of job appeal, with higher scores indicating higher job appeal (masculine α = 0.88, feminine α = 0.87). Two choice questions also measured job appeal, asking participants “*Which job would you be most likely to want?/enjoy?”*

#### Job Fit

Job fit was measured using 4 items (adapted from Gaucher et al., 2011) asking participants “*please indicate to what extent you agree or disagree with the following statements… I could fit in well at this company/ I'm similar to the people who work in this company/ My values and this company's values are similar/ The type of people who would apply for this job are very different from me.”* A mean score was used as the index of job appeal, with higher scores indicating higher job fit (masculine α = 0.91, feminine α = 0.89). Two choice questions also measured job fit, asking participants “*Which job would be the best fit for you?/ The people at which company do you think would be most similar to you?”*

#### Self-Rated Leadership Potential

Self-rated leadership potential was measured using 7 items asking participants “*to what extent to you agree or disagree that the [company name] offers you the opportunity to fulfill your… leadership potential/ potential to become a successful leader/ capability to become a successful leader, potential to develop leadership skills/ potential to advance to a leadership position/ potential to become a leader is a role-model for your co-workers”* The items were as in Study 1. A mean score was used as the index of self-rated leadership potential, with higher scores indicating higher leadership potential (masculine α = 0.95, feminine α = 0.95). Two choice questions also measured leadership potential, asking participants “*Which job can help you fulfill your potential to be a successful leader?/ Which job can help you fulfill your potential to advance to a leadership position?”*^5^.

### Results

To test the interaction between gender and gender-stereotyped organizational culture on self-rated leadership potential for men and women, we conducted a repeated-measures ANOVA with gender (men vs. women) as a between-participants variables and workplace culture (masculine, feminine) as the within-participants variable.

#### Manipulation Checks

##### Organizational culture and gender stereotypes

There was a significant main effect of organizational culture on perceptions of women enjoying the role, *F*_(1, 197)_ = 14.77, *p* < 0.001, η^2^ = 0.07. Participants perceived that women would be more likely to enjoy the feminine organizational culture (*M* = 5.38, *SD* = 1.10) than the masculine organizational culture (*M* = 5.12, *SD* = 1.16). There was no main effect of gender [*F*_(1, 197)_ = 0.37, *p* = 0.54, η^2^ = 0.002] and no interaction effect [*F*_(1, 197)_ = 0.02, *p* = 0.88, η^2^ < 0.001].

There was no main effect of organizational culture on perceptions of men enjoying the role [*F*_(1, 197)_ = 0.16, *p* = 0.69, η^2^ = 0.001]. There was no main effect of gender [*F*_(1, 197)_ = 0.10, *p* = 0.76, η^2^ < 0.001] and no interaction effect [*F*_(1, 197)_ = 0.07, *p* = 0.80, η^2^ < 0.001].

### Dependent Measures

#### Job appeal

There was a significant main effect of organizational culture on job appeal, *F*_(1, 197)_ = 37.93, *p* < 0.001, η^2^ = 0.16. The job in the feminine organizational culture was perceived to be more appealing (*M* = 4.75, *SD* = 1.07) than the job in the masculine organizational culture (*M* = 4.29, *SD* = 1.12). There was no main effect of participant gender on job appeal [*F*_(1, 197)_ = 0.76, *p* = 0.39, η^2^ = 0.004] and no interaction effect [*F*_(1, 197)_ = 0.59, *p* = 0.45, η^2^ = 0.003]. All means and standard deviations for Study 2 are reported in Table 5 and correlations in Table 6.

**Table 5:** Study 2: means and standard deviations for job appeal, job fit, and self-rated leadership potential.

	**Male participants**		**Female participants**	
	**Masculine**	**Feminine**	**Masculine**	**Feminine**
	**M(SD)**	**M(SD)**	**M(SD)**	**M(SD)**
Job appeal	4.26 (1.08)	4.66 (1.03)	4.32 (1.17)	4.84 (1.10)
Job fit	3.96 (1.23)	4.34 (1.15)	3.87 (1.26)	4.53 (1.18)
Leadership potential	5.10 (1.08)	5.27 (0.94)	5.30 (0.91)	5.59 (0.78)

**Table 6:** Study 2: correlation matrix for job appeal (agentic and communal), job fit (agentic and communal), and self-rated leadership potential (agentic and communal).

	**1**	**2**	**3**	**4**	**5**	**6**
1. Job appeal (agentic)		0.54^**^	0.86^**^	0.49^**^	0.39^**^	0.24^**^
2. Job appeal (communal)			0.48^**^	0.79^**^	0.31^**^	0.45^**^
3. Job fit (agentic)				0.57^**^	0.24^**^	0.13
4. Job fit (communal)					0.11	0.24^**^
5. Leadership potential (agentic)						0.65^**^
6. Leadership potential (communal)						

** *Correlation significant at the 0.01 level (2-tailed)*.

There was no association between gender and wanting the job in either culture [${\text{χ}}_{\left(1,\;\text{N}=199\right)}^{2}$ = 0.69, *p* = 0.41]. There was no association between gender and perceptions of enjoying the job in either culture [${\text{χ}}_{\left(1,\;\text{N}=199\right)}^{2}$ = 0.05, *p* = 0.83].

#### Job fit

There was a significant main effect of organizational culture on perceived fit, *F*_(1, 197)_ = 43.12, *p* < 0.001, η^2^ = 0.18. The feminine organizational culture was perceived to be higher fit (*M* = 4.44, *SD* = 1.16) than the masculine organizational culture (*M* = 3.91, *SD* = 1.24). There was no main effect of participant gender on perceived fit [*F*_(1, 197)_ = 0.10, *p* = 0.76, η^2^ < 0.001].

The main effect of organizational culture was qualified by a marginally-significant interaction between organizational culture and participant gender, *F*_(1, 197)_ = 2.96, *p* = 0.09, η^2^ = 0.02. Further analyses showed that women perceived more fit in the feminine organizational culture (*M* = 4.53, *SD* = 1.18) than the masculine organizational culture (*M* = 3.87, *SD* = 1.26); *F*_(1, 197)_ = 36.34, *p* < 0.001, η^2^ = 0.16. Men also perceived more fit in the feminine organizational culture (*M* = 4.34, *SD* = 1.15) than the masculine organizational culture (*M* = 3.96, *SD* = 1.23); *F*_(1, 197)_ = 11.13, *p* = 0.001, η^2^ = 0.05, although to a lesser extent than women.

There was no association between gender and perceiving a better fit either culture [${\text{χ}}_{\left(1,\;\text{N}=199\right)}^{2}$ = 0.98, *p* = 0.32]. There was no association between gender and perceptions of similarity to other people in the company in either culture [${\text{χ}}_{\left(1,\;\text{N}=199\right)}^{2}$ = 0.69, *p* = 0.41].

#### Self-Rated leadership potential

There was a significant main effect of organizational culture on self-rated leadership potential, *F*_(1, 197)_ = 17.63, *p* < 0.001, η^2^ = 0.08. Participants self-rated higher leadership potential in the feminine organizational culture (*M* = 5.44, *SD* = 0.87) than the masculine organizational culture (*M* = 5.20, *SD* = 1.00). There was a significant main effect of participant gender on self-rated leadership potential, *F*_(1, 197)_ = 4.76, *p* = 0.03, η^2^ = 0.02. Women self-rated higher leadership potential (*M* = 5.45, *SD* = 0.08) than men (*M* = 5.19, *SD* = 0.09). There was no interaction effect between organizational culture and participant gender on self-rated leadership potential [*F*_(1, 197)_ = 1.18, *p* = 0.28, η^2^ = 0.01].

There was no association between gender and perceptions of fulfilling potential in either culture [${\text{χ}}_{\left(1,\;\text{N}=199\right)}^{2}$ = 1.75, *p* = 0.19]. There was no association between gender and perceptions of advancing to a leadership position in either culture [${\text{χ}}_{\left(1,\;\text{N}=199\right)}^{2}$ = 0.53, *p* = 0.47].

### Intersectional Analyses

To test the intersectional effects of gender stereotyped organizational culture on self-rated leadership potential, we conducted an exploratory analysis using participant age as a continuous moderator (using model 1 in PROCESS, Hayes, 2013). We introduced gender as the predictor, participant age as a continuous moderator variable. To test the interaction of participant gender and participant age on the dependent variables (job appeal in models 1–2, job fit in models 3–4, and self-rated leadership potential in models 5–6), we ran the moderations independently for each culture (masculine culture in models 1, 3, 5 and feminine culture in models 2, 4, 6).

#### Job Appeal

In model 1, there was no main effect of gender (*b* = −0.74, *SE* = 0.70, *t* = −1.06, *p* = 0.29, 95% CI −2.13, 0.64) or age (*b* = −0.04, *SE* = 0.03, *t* = −1.33, *p* = 0.18, 95% CI −0.10, 0.02), and no interaction of gender and age (*b* = 0.02, *SE* = 0.02, *t* = 1.17, *p* = 0.24, 95% CI −0.02, 0.06) on job appeal in the masculine organizational culture.

In model 2, there was no main effect of gender (*b* = −0.66, *SE* = 0.67, *t* = −0.99, *p* = 0.32, 95% CI −1.98, 0.66) or age (*b* = −0.04, *SE* = 0.03, *t* = −1.19, *p* = 0.24, 95% CI −0.09, 0.02), and no interaction of gender and age (*b* = 0.02, *SE* = 0.02, *t* = 1.29, *p* = 0.20, 95% CI −0.01, 0.06) on job appeal in the feminine organizational culture.

#### Job fit

In model 3, there was no main effect of gender (*b* = −0.47, *SE* = 0.78, *t* = −0.60, *p* = 0.55, 95% CI −2.00, 1.07) or age (*b* = −0.02, *SE* = 0.03, *t* = −0.61, *p* = 0.54, 95% CI −0.09, 0.05), and no interaction between gender and age (*b* = 0.01, *SE* = 0.02, *t* = 0.49, *p* = 0.62, 95% CI −0.03, 0.05) on perceptions of job fit in the masculine organizational culture.

In model 4, there was no main effect of gender (*b* = −0.62, *SE* = 0.72, *t* = −0.86, *p* = 0.39, 95% CI −2.05, 0.81) or age (*b* = −0.03, *SE* = 0.03, *t* = −0.84, *p* = 0.40, 95% CI −0.09, 0.04), and no interaction of gender and age (b = 0.02, *SE* = 0.02, t = 1.15, p = 0.25, 95% CI −0.02, 0.06) on perceptions of job fit in the feminine organizational culture.

#### Self-Rated leadership potential

In model 5, there was no main effect of gender (*b* = −0.62, *SE* = 0.62, *t* = −1.00, *p* = 0.32, 95% CI −1.84, 0.60) or age (*b* = −0.04, *SE* = 0.03, *t* = −1.55, *p* = 0.12, 95% CI −0.10, 0.01), and no interaction effect between gender and age (*b* = 0.02, *SE* = 0.02, *t* = 1.35, *p* = 0.18, 95% CI −0.01, 0.06) on self-rated leadership potential in the masculine organizational culture.

In model 6, there was no main effect of gender (*b* = −0.17, *SE* = 0.54, *t* = −0.31, *p* = 0.75, 95% CI −1.23, 0.89), no main effect of age (*b* = −0.02, *SE* = 0.02, *t* = −0.75, *p* = 0.45, 95% CI −0.06, 0.03), and no interaction of gender and age (*b* = 0.01, *SE* = 0.01, *t* = 0.94, *p* = 0.35, 95%CI −0.01, 0.04) on self-rated leadership potential in the feminine organizational culture.

### Discussion

We found two interesting and unexpected findings in Study 2. First, there was an overall preference for the feminine organizational culture across both genders. This finding partially supports Hypothesis 5, women preferred the feminine organizational culture, but men's preference for the feminine organizational culture was not hypothesized. This preference may reflect a general orientation toward less hierarchical and more cooperative workplace cultures in recognition of the benefits this can bring in terms of performance and commitment (e.g., Triguero-Sánchez et al., 2018).

Second, and contrary to Hypothesis 1, women self-rated higher leadership potential than men. This reflects the nature of the findings in Study 1- women do not rate less leadership potential in themselves compared to men. Although assessments of leadership potential in others are affected by gender to the detriment of women (Tresh et al., 2018; Player et al., in press), self-assessments of leadership potential for younger women are not—at least directly. The limited age range of our participants may provide an explanation for why we did not replicate the intersectional effects found in Study 1. Further research is warranted with a representative sample of older people to determine these effects.

Organizational culture had the biggest impact on women in terms of perceived fit—although both women and men perceived more fit in the feminine organizational culture, this difference was greater for women. Our conclusion can be framed positively—even when women perceive low organizational fit, this does not reduce their self-rated of leadership potential. However, research on women's career progression suggests that low organizational fit inhibits access to informal networks, inclusion, and stretch opportunities (Simpson, 2000). Nonetheless, high self-rated leadership potential in younger women may reflect the apparent leadership advantage and disadvantage—the progress made toward gender equality coupled with lack of achievement in fully reaching it (Eagly, 2007).

Overall, the findings of Study 2 partially support our hypotheses. Women, and also men, prefer a stereotypically feminine organizational culture. Women self-rated higher leadership potential than men, though the intersectional nature of this should be determined.

## Study Three

In Study 1 we found that men and younger people are more likely to endorse the “beneficial” stereotypes that relate them more closely to leadership than women and/ or their older counterparts. Whereas, endorsing gender stereotypes did not interact to predict self-rated leadership potential for younger women, both gender and age stereotypes interacted to predict self-rated leadership potential for older women. Furthermore, older men are potentially negatively affected in terms of self-rated leadership potential by the warmth attributed to older people. In this study, we extend societal stereotypes to reinforced workplace stereotypes to test Hypothesis 6.

### Method

#### Participants and Design

We recruited 217 participants through Prolific. After removing 28 participants for the reasons outlined in study 1, no participants were removed based on their responses to manipulation checks, and 189 participants were retained and their data used in the analyses presented below (49 men, 140 women, 18–65 years; *M* = 40.97, *SD* = 15.17). There were 93 participants in the younger group (*M* = 26.05, *SD* = 3.08) and 96 participants in the older group (*M* = 55.43, *SD* = 4.16). All participants were in full- or part-time employment. Participants were paid £0.95 for taking part in the online study and the average completion time was 382.03 (*SD* = 146.14).

The Study adopted a 2 Participant age (younger vs. older) x 2 Workplace culture (younger, older) quasi-experimental mixed design. Participant age was a between-participants variable, whereas workplace culture was a within-participants variable. Dependent variables measured job appeal, job fit, and self-rated leadership potential.

### Procedure and Materials

The procedure for study three mirrored that for study two. The only difference was the manipulation of workplace culture, which was operationalized to reflect younger and older workplace cultures rather than masculine and feminine cultures.

The descriptors used in the younger workplace condition were: *keen, energetic, ambitious, willing to learn*, and *fast learner*; those used for the older workplace condition were *experienced, mature, knowledgeable, professional*, and *provides stability*. Descriptors were sourced from the existing literature (Posthuma and Campion, 2009; Swift et al., 2013; Abrams et al., 2016).

### Measures

Questions were scored on a seven-point scale (1 = *very much disagree*, 7 = *very much agree*), with the exception of choice questions.

#### Manipulation Checks

The measures adopted in study three replicate those used in study two, referring to age (i.e., “younger people” or “older people”) instead of gender. Therefore, the same items were used for the manipulation checks: “*Think about this job advert and please indicate the extent to which you agree with the following… younger people would enjoy this job/ older people would enjoy this job.”*

#### Dependent Measures

Measures of job appeal (younger culture α = 0.89; older culture α = 0.88), job fit (younger culture α = 88; older culture α = 0.87), and self-rated leadership potential (younger culture α = 0.96; older culture α = 0.96) were the same as those used in study two^6^.

### Results

To test the interaction between age and age-stereotyped organizational culture on self-rated leadership potential for younger and older workers, we conducted a repeated-measures ANOVA with age (younger workers vs. older workers) as a between-participants variables and workplace culture (younger, older) as the within-participants variable.

#### Manipulation Checks

##### Organizational culture and age stereotypes

There was a significant main effect of organizational culture on perceptions of younger workers enjoying the role, *F*_(1, 187)_ = 30.97, *p* < 0.001, η^2^ = 0.14. Participants perceived younger people to be more likely to enjoy the younger organizational culture (*M* = 5.25, *SD* = 1.27) than the older organizational culture (*M* = 4.66, *SD* = 1.21). There was a significant main effect of participant age on perceptions of younger workers enjoying the role, *F*_(1, 187)_ = 8.03, *p* = 0.005, η^2^ = 0.04. Participants were more likely to perceive that younger people would enjoy this role, if they were older workers (*M* = 5.16, *SD* = 0.10) compared to younger workers (*M* = 4.75, *SD* = 0.10). There was no interaction effect [*F*_(1, 187)_ = 0.41, *p* = 0.52, η^2^ = 0.002].

There was a significant main effect of organizational culture on perceptions of older people enjoying the role, *F*_(1, 187)_ = 36.33, *p* < 0.001, η^2^ = 0.16. Participants perceived older people to be more likely to enjoy the older organizational culture (*M* = 4.80, *SD* = 1.21) than the younger organizational culture (*M* = 4.18, *SD* = 1.45). There was no main effect of participant age [*F*_(1, 187)_ = 2.00, *p* = 0.16, η^2^ = 0.01], and no interaction effect [*F*_(1, 187)_ = 0.003, *p* = 0.95, η^2^ < 0.001].

#### Dependent Measures

##### Job appeal

There was no main effect of organizational culture on job appeal [*F*_(1, 187)_ = 0.50, *p* = 0.48, η^2^ = 0.003]. There was no main effect of participant age on job appeal [*F*_(1, 187)_ = 1.45, *p* = 0.23, η^2^ = 0.01]. There was a significant interaction between organizational culture and participant age on job appeal, *F*_(1, 187)_ = 10.01, *p* = 0.002, η^2^ = 0.05.

Further analyses showed that in the younger organizational culture younger workers perceived the job to be more appealing (*M* = 4.67, *SD* = 1.22) than older workers (*M* = 4.25, *SD* = 1.16), *F*_(1, 187)_ = 5.77, *p* = 0.02, η^2^ = 0.03. There was no difference in the older organizational culture [*F*_(1, 187)_ = 0.11, *p* = 0.74, η^2^ = 0.001]. Older people perceived the job in the older organizational culture to be more appealing (*M* = 4.54, *SD* = 1.09) than in the younger organizational culture (*M* = 4.25, *SD* = 1.16), *F*_(1, 187)_ = 7.60, *p* = 0.006, η^2^ = 0.04. Younger people did not perceive the job in either culture as significantly more appealing than the other [*F*_(1, 187)_ = 2.98, *p* = 0.09, η^2^ = 0.02]. All means and standard deviations for Study 3 are reported in Table 7 and correlations in Table 8.

**Table 7:** Study 3: means and standard deviations for job appeal, job fit, and self-rated leadership potential.

	**Younger participants**		**Older participants**	
	**Younger culture**	**Older culture**	**Younger culture**	**Older culture**
	**M(SD)**	**M(SD)**	**M(SD)**	**M(SD)**
Job appeal	4.66 (1.22)	4.48 (1.14)	4.25 (1.16)	4.54 (1.09)
Job fit	4.20 (1.25)	4.12 (1.11)	3.82 (1.25)	4.24 (1.20)
Leadership potential	5.23 (1.21)	5.24 (1.15)	5.42 (0.93)	5.44 (0.88)

**Table 8:** Study 3: correlation matrix for all dependent variables.

	**1**	**2**	**3**	**4**	**5**	**6**
1. Job appeal (younger culture)		0.60^**^	0.81^**^	0.57^**^	0.59^**^	0.42^**^
2. Job appeal (older culture)			0.54^**^	0.83^**^	0.41^**^	0.54^**^
3. Job fit (younger culture)				0.62^**^	0.41^**^	0.26^**^
4. Job fit (older culture)					0.35^**^	0.44^**^
5. Leadership potential (younger culture)						0.70^**^
6. Leadership potential (older culture)						

** *Correlation significant at the 0.01 level (2-tailed)*.

There was a significant association between age and wanting the job in either culture, ${\text{χ}}_{\left(1,\;\text{N}=189\right)}^{2}$ = 6.47, *p* = 0.01. Specifically, older people were more likely to want the job in the older organizational culture (66.7%) than the younger organizational culture (33.3%). There was a significant association between age and perceptions of enjoying the job in either culture, ${\text{χ}}_{\left(1,\;\text{N}=189\right)}^{2}$ = 13.72, *p* < 0.001. Specifically, older people were more likely to perceive that they would enjoy the job in the older organizational culture (65.6%) than the younger organizational culture (34.4%).

##### Job fit

There was a main effect of organizational culture on perceived fit, *F*_(1, 187)_ = 5.14, *p* = 0.02, η^2^ = 0.03. The older organizational culture was perceived to be higher fit (*M* = 4.18, *SD* = 1.16) than the younger organizational culture (*M* = 4.01, *SD* = 1.26). There was no main effect of participant age on perceived fit [*F*_(1, 187)_ = 0.65, *p* = 0.42, η^2^ = 0.003]. There was a significant interaction between organizational culture and participant age on perceived fit, *F*_(1, 187)_ = 11.16, *p* = 0.001, η^2^ = 0.06.

Further analyses showed that in the younger organizational culture younger workers perceived greater organizational fit (*M* = 4.20, *SD* = 1.25) than older workers (*M* = 3.82, *SD* = 1.25), *F*_(1, 187)_ = 4.36, *p* = 0.04, η^2^ = 0.02. There was no difference in the older organizational culture [*F*_(1, 187)_ = 0.54, *p* = 0.46, η^2^ = 0.003]. Older people perceived greater organizational fit in the older organizational culture (*M* = 4.25, *SD* = 1.20) than in the younger organizational culture (*M* = 3.82, *SD* = 1.25), *F*_(1, 187)_ = 15.98, *p* < 0.001, η^2^ = 0.08. Younger people did not perceive greater organizational fit in either culture [*F*_(1, 187)_ = 0.57, *p* = 0.45, η^2^ = 0.003].

There was a significant association between age and perceiving a better fit either culture, ${\text{χ}}_{\left(1,\text{N}=189\right)}^{2}$ = 8.05, *p* = 0.005. Specifically, older people were more likely to perceive a better fit in the older organizational culture (66.7%) than the younger organizational culture (32.3%). There was a significant association between age and perceptions of similarity to other people in the company in either culture, ${\text{χ}}_{\left(1,\text{N}=189\right)}^{2}$ = 5.75, *p* = 0.02. Specifically, older people were more likely to perceive similarity to people in the older organizational culture (66.7%) than the younger organizational culture (33.3%).

##### Self-Rated leadership potential

There was no main effect of organizational culture on self-rated leadership potential [*F*_(1, 187)_ = 0.08, *p* = 0.77, η^2^ < 0.001]. There was no main effect of participant age on self-rated leadership potential [*F*_(1, 187)_ = 2.03, *p* = 0.16, η^2^ = 0.01. There was no interaction effect between organizational culture and participant age on self-rated leadership potential [*F*_(1, 187)_ = 0.02, *p* = 0.89, η^2^ < 0.001].

There was no association between age and perceptions of fulfilling potential in either culture [${\text{χ}}_{\left(1,\text{N}=189\right)}^{2}$ = 1.50, *p* = 0.22]. There was no association between age and perceptions of advancing to a leadership position in either culture [${\text{χ}}_{\left(1,\text{N}=189\right)}^{2}$ = 0.41, *p* = 0.52].

#### Intersectional Analyses

To test the intersectional effects of age stereotyped organizational culture on self-rated leadership potential, we conducted an exploratory analysis using repeated-measures ANOVA with age and gender as between-participants variables and workplace culture as the within-participants variable. This resulted in 28 younger men, 65 younger women, 21 older men, and 75 older women.

##### Job appeal

There was no main effect of participant gender on job appeal [*F*_(1, 185)_ = 0.69, *p* = 0.41, η^2^ = 0.004]. There was no interaction effect between participant age and participant gender on job appeal [*F*_(1, 185)_ = 1.55, *p* = 0.21, η^2^ = 0.01]. There was no interaction effect between organizational culture and participant gender on job appeal [*F*_(1, 185)_ = 0.02, *p* = 0.89, η^2^ < 0.001]. There was no three-way interaction between participant age, participant gender and organizational culture on job appeal [*F*_(1, 185)_ = 0.35, *p* = 0.56, η^2^ = 0.002].

##### Job fit

There was no main effect of participant gender on perceived fit [*F*_(1, 185)_ = 0.26, *p* = 0.61, η^2^ = 0.001]. There was no interaction effect between participant age and participant gender on perceived fit [*F*_(1, 185)_ = 0.88, *p* = 0.35, η^2^ = 0.01]. However, there was a significant interaction effect between organizational culture and participant gender on perceived fit; *F*_(1, 185)_ = 8.46, *p* < 0.005, η^2^ = 0.04. Further analyses showed that women perceived greater organizational fit in the older organizational culture (*M* = 4.28, *SD* = 1.14) than the younger organizational culture (*M* = 3.98, *SD* = 1.25), *F*_(1, 185)_ = 12.13, *p* = 0.001, η^2^ = 0.06. Men perceived no difference in organization fit between the cultures [*F*_(1, 185)_ = 1.77, *p* = 0.19, η^2^ = 0.01]. There was no three-way interaction between participant age, participant gender and organizational culture on perceived fit [*F*_(1, 185)_ = 0.01, *p* = 0.93, η^2^ < 0.001].

##### Self-rated leadership potential

There was a significant main effect of participant gender on self-rated leadership potential, *F*_(1, 185)_ = 5.23, *p* = 0.02, η^2^ = 0.03. Women had higher self-rated leadership potential (*M* = 5.44, *SD* = 0.08) than men (*M* = 5.07, *SD* = 0.14). There was no interaction effect between participant age and participant gender on self-rated leadership potential [*F*_(1, 185)_ = 1.81, *p* = 0.18, η^2^ = 0.01]. There was no interaction effect between organizational culture and participant gender on self-rated leadership potential [*F*_(1, 185)_ = 0.04, *p* = 0.85, η^2^ < 0.001]. There was no three-way interaction between participant age, participant gender and organizational culture on self-rated leadership potential [*F*_(1, 185)_ = 0.08, *p* = 0.78, η^2^ < 0.001].

### Discussion

Contrary to hypothesis 3, we found no relationship between age and self-rated leadership potential in this study. Furthermore, we found no interaction effects between organizational culture and participant age on self-rated leadership potential. There are two possible reasons for this. First, this could suggest that endorsing in-group stereotypes has a stronger effect than reinforcing stereotyped organizational culture on self-rated leadership potential. Stereotype embodiment theory (Levy, 2009) argues that age stereotypes are assimilated from the surrounding culture from childhood, and so it may be that the general societal context has more influence on age-based stereotyped thinking than the specific organizational context. Our hypotheses about the strength of effects for endorsement (of societal stereotypes) vs. reinforcement (of organizational culture) warrants further investigation. Second, the non-significant findings for self-rated leadership potential could also reflect a difference in the impact of different types of age-based stereotypes. The warmth-competence stereotype characteristics used in Study 1 may have greater influence on self-rated leadership potential than the alternative age stereotype characteristics used in Study 3; this potential difference could be addressed further in future research.

In support of Hypothesis 6, we observed interaction effects for job appeal and job fit, replicating our findings on the effects of stereotyped organizational culture on women's perceptions of organizational fit as found in Study 2. Additionally in this study, older workers, unlike women, also found the age-congruent organizational culture more appealing. The greater impact of workplace stereotypes on older workers compared to women may reflect the difference between gender and age stereotypes—gender is (mostly) fixed whereas age is fluid, and so negative older-age stereotypes become more self-relevant as people age. For instance, older workers may not necessarily perceive themselves in line with old-age stereotypes (stereotypes they perceive to be directed at *other* older people, Swift et al., 2017). Research suggests that old-age stereotypes have to be self-relevant in order to have a detrimental effect on either attitudes or behavior (Levy, 2009; Marques et al., 2014), which could explain why the age-typed organizational culture affected older workers perceptions of job appeal.

The inclusion of gender as a potentially intersecting identity did not yield the intersectional effects found in Studies 1 and 2. Gender did not interact with age and organizational culture to show a greater impact of organizational culture on older women compared to older men. However, this could reflect the nature of descriptors used in Study 3 compared to Study 1.

## General Discussion

Overall, we found partial support for our hypotheses across three studies. With respect to gender, and contrary to Hypothesis 1, we found that women self-rate the same amount (Study 1), if not more (Study 2), leadership potential than men. However, women's ratings of job fit are influenced by organizational culture (Hypothesis 5). With respect to age, we found a relationship between age and self-rated leadership potential (Hypothesis 3) in Study 1 but not Study 3. Furthermore, organizational culture impacts older workers' perceptions of job fit and job appeal (Hypothesis 6).

An important finding relates to the exploratory results on the inter-sectionality of gender and age in Study 1. We found evidence that both gender and age stereotypes have a greater impact on older people compared to younger people. We also found that age stereotypes relate to self-rated leadership potential differently for older men and older women. Namely, endorsing competency-based age stereotypes reduced self-rated leadership potential in older women but not older men. Endorsing warmth-based stereotypes reduced self-rated leadership potential in older men but not older women. Nonetheless, endorsing the “softer” stereotypical traits of women was in fact positively related to older women's self-rated leadership potential. Notably, and perhaps unsurprisingly, younger men's self-rated leadership potential was advantaged by endorsing gender stereotypes about women being more communal than men. Surprisingly, younger women's self-rated leadership potential was not related to endorsing any stereotypes, which might reflect generational effects.

The more pronounced effects for older people compared to younger people may be explained by older people's lack of opportunity for leaving their low status group compared to younger people (Garstka et al., 2004). This is particularly likely for older women who are stereotypically leadership-incongruent based on their gender and age. We examined the effects for older people in Study 3. Older people self-rated less job appeal and less job fit in the younger culture. The limited representation of older people in Study 2 made it difficult to draw conclusions about the intersectional effects, particularly for older women. Also, the limited representation of men in Study 3 also made examining the intersectional effects more challenging. Based on the intersectional effects observed in Study 1, these warrant further investigation.

## Theoretical and Applied Implications

We contribute to three areas of research in social psychology. First, we extend research on stereotypes to reveal the impact of societal and organizational stereotypes on gender and age groups. Second, we introduce a new perspective for examining the subjectivity of leadership potential, looking at antecedents of self-rated leadership potential as opposed to evaluators' perceptions of a target's leadership potential. Finally, we shed light on the effects of stereotypes at the intersectional level of gender and age.

We contribute to the stereotypes literature by highlighting the significant and interactive role of agentic and communal gender stereotypes and competence and warmth age stereotypes. The findings suggest that, it may not be endorsing the stereotype that women and older people are too communal or warm, it may instead be the focus on the extent to which the stereotype is leader (in)congruent which relates to self-rated leadership potential. We cannot however generalize these findings to evaluator-driven research and instead conclude that this has been found in our target-driven research.

Research has shown that the gender gap is greater in masculine-stereotyped domains, evidencing an effect of stereotyped organizational culture on evaluators' perceptions (Elesser and Lever, 2011). We did not find this for self-ratings of leadership potential, however, we did find this for self-rated organizational fit. We cannot determine from our data whether societal stereotypes or stereotyped organizational cultures have more impact on women in general, because women self-rated as much, and more, leadership potential than men. However, the impact of societal stereotypes may in fact be more detrimental than organizational stereotypes because they may be likely to have more of an impact at earlier stages of women's careers. For example, endorsing stereotypes that disassociate women from leadership may discourage women from prioritizing leadership attainment in career planning. For women who are not deterred from pursuing leadership roles, their leadership ambition may counteract perceptions of organizational fit to pursue their potential to lead. This rationale would certainly support our findings. For older people, particularly older women, the role of societal stereotypes likely affects the extent to which they feel efficacy to change fields or train in an alternate profession. Research has documented the immediate effects of receiving feedback about one's leadership potential on performance and ambition (Steffens et al., 2018). Thus, frequent career cues such as workplace stereotypes, potential and performance evaluations are likely to have a stronger impact than job advertisements.

Our exploratory analyses at the intersectional level demonstrates the complex ways in which stereotypes can influence their targets. The results of our research demonstrating that younger women are less affected by gender stereotypes than older women highlight the need for further research to examine other high-level and low-level intersecting identities. What impact do gender and racial stereotypes have on minority ethnic women's perceptions of their own leadership potential in comparison to white women? It is likely that, as with older women, the detrimental effects for minority ethnic women are more prominent (Mirza, 2003). This warrants further empirical exploration and emphasizes the need for organizations to address diversity and equality as issues of intersectionality that have otherwise focused on white women for gender initiatives and black men for race initiatives (Ghavami and Peplau, 2013).

## Limitations and Future Directions

Our first study demonstrated clear intersectional interactions on self-rated leadership potential for older women in particular. However, the cross-sectional data of this study limits our conclusions. Although we expect that endorsing leadership-incongruent stereotypes about one's identity would reduce self-rated leadership potential, we cannot conclude the causal nature of this relationship. Studies 2 and 3 provide experimental tests, but future research should continue to uncover the underlying causal mechanisms with representative groups across ages and gender to examine the intersectional effects with A-priori hypotheses.

Studies 2 and 3 used experimental vignettes to manipulate workplace culture. There is evidence of a relationship between vignettes and real-life situations (e.g., Ganong and Coleman, 2005, 2006), and experimental vignettes are regarded as a reliable method of exploring research topics whilst maintaining control of the research process (Doz, 2011). Nonetheless, and given the novel findings, further research would benefit from complementary studies which consider the workplace cultures that people experience on a daily basis. We also designed our experiments to investigate the effects of gender stereotypes and age stereotypes independently, this should be considered in relation to the inclusion of our intersectional exploratory analysis, where the older age category could be better represented in Study 2. It is possible that participants were aware of the nature of our hypotheses, as endorsement of stereotypes were asked explicitly in Study 1 and the within-participants design of Studies 2 and 3 could allow for comparisons to be drawn easily between the two organizational cultures. We recommend the use of deception checks for future research.

Our hypotheses were such that identity-incongruent stereotyped organizational cultures would reduce women's and older people's self-rated leadership potential. We did not support these hypotheses and also did not determine causes of these null effects. In Study 1 we measured endorsement of stereotypes, and in Studies 2 and 3 we manipulated stereotyped organizational culture. Therefore, we have not directly compared the effects of stereotype endorsement vs. stereotype reinforcement. Future research should address two limitations. First, experimentally compare societal stereotypes and organizational culture to identify which has greater effects on self-rated leadership potential, or if there is an interactive effect. Second, examine why, given stereotyped organizational culture affects women's and older people's perceptions of organizational fit, it does not have detrimental outcomes for self-rated leadership potential.

Finally, we limited our examination of the role of stereotypes on women and older people's self-rated leadership potential. Stereotypes are likely to have similar effects for other protected characteristics such as ethnicity, disability, particularly at the intersectional level also for other potential factors (e.g., maternity/paternity). Research should focus on identifying the leadership-related stereotypes that hinder the progression of underrepresented groups such as ethnic minorities (Gündemir et al., 2014) to drive forward research on understanding the internal barriers members of groups with protected characteristics face in leadership attainment.

## Conclusion

We have contributed to a growing interest in the impact of bias on the targets of prejudice and the social-psychological variables contributing to the subjective nature of leadership potential. If women and older people are to be perceived as future leaders by others, they should first be able to perceive themselves as future leaders, without the constraints of societal stereotypes. Overall, promising findings indicate that stereotypes may be having less impact on younger women than their older counterparts.

## Ethics Statement

All experiments were carried out in accordance with the recommendations of the School of Psychology Ethics Committee at the University of Kent, United Kingdom. The protocol was approved by the School of Psychology Ethics Committee. All participants gave written informed consent in accordance with the Declaration of Helsinki. The research was conducted in accordance with guidelines from the University of Kent Research Ethics (Human Participants) Committee, the Economic and Social Research Council (ESRC) Research Ethics Framework, and the ethical guidelines from the British Psychology Society (BPS).

## Author Contributions

FT and BS conceived the presented research hypotheses and design. GR initiated the collaborative study of target group members' perceptions of their own leadership potential. GR and AL provided theory development and feedback on research designs. FT led on the data analysis and writing of the paper, supported by BS in both respects. GR and AL advised on analytical methods and provided critical feedback on drafts of the paper. All authors helped shape the overall research, with research ideas partially grounded in earlier work by HS and AP.

### Conflict of Interest Statement

The authors declare that the research was conducted in the absence of any commercial or financial relationships that could be construed as a potential conflict of interest.

## Footnotes

^1^Two participants indicated “other gender” however, given the gender intergroup nature of the study and the lack of representation of non-binary categories, we did not include these two participants in the analyses reported below.

^2^Three participants fell outside of the range of the two age categories and were not included in the analysis reported below.

^3^Results for the ‘adaptability' dimension of the adapted ‘work-related age-based stereotypes scale' are available upon request.

^4^We measured perceptions of access to development opportunities, reliability of the scale and the relationship with other variables are available upon request.

^5^We also measured leadership aspirations, reliability of the scale and the relationship with other variables are available upon request.

^6^We also measured leadership aspirations, reliability of the scale and the relationship with other variables are available upon request.

## References

[B1] Abrams D. Swift H. J. Drury L. (2016). Old and unemployable? How age-based stereotypes affect willingness to hire job candidates. J. Soc. Issues 72, 105–121. 10.1111/josi.12158 27635102 PMC4999032

[B2] Atewologun D. Cornish T. Tresh F. (2018). Unconscious Bias Training: An Assessment if the Evidence for Effectiveness. Equality and Human Rights Commission Research Report Series.

[B3] Bantel K. A. Jackson S. E. (1989). Top management and innovations in banking: does the composition of the top team make a difference? Strategic Manag. J. 10, 107–124. 10.1002/smj.4250100709

[B4] Beck V. Williams G. (2016). Managing Older Workers: A Report for ACAS (research paper 05/16). Retrieved from ACAS website: http://www.acas.org.uk/media/pdf/f/i/Managing-older-workers-a-report-for-acas.pdf (accessed March 22, 2019).

[B5] Bem S. L. (1981). Gender schema theory: a cognitive account of sex typing. Psychol. Rev. 88, 354–364. 10.1037/0033-295X.88.4.354

[B6] Bettencourt B. Charlton K. Dorr N. Hume D. L. (2001). Status differences and in-group bias: a meta-analytic examination of the effects of status stability, status legitimacy, and group permeability. Psychol. Bull. 127, 520–542. 10.1037//0033-2909.127.4.520 11439710

[B7] Bowles H. R. Babcock L. McGinn K. L. (2005). Constraints and triggers: situational mechanics of gender in negotiation. J. Pers. Soc. Psychol. 89:951. 10.1037/0022-3514.89.6.951 16393027

[B8] Broadbridge A. (2001). Ageism in retailing: myth or reality?, in Ageism in Work and Employment, eds Golver I. Branine M. (Burlington, VT: Ashgate), 153–174.

[B9] Business in the Community (2016). Age in the Workplace: Retain, Retrain, Recruit. Retrieved from: https://age.bitc.org.uk/all-resources/research-articles/age-workplace-retain-retrain-recruit (accessed March 22, 2019).

[B10] Business in the Community (2017). Government Publish New Labour Market Statistics on Over 50's. Retrieved from: https://age.bitc.org.uk/news-opinion/news/government-publish-new-labour-market-statistics-over-50s (accessed March 22, 2019).

[B11] Catalyst (2018). Quick Take: Women in the Workforce. Retrieved from: https://www.catalyst.org/knowledge/women-workforce-uk (accessed March 22, 2019).

[B12] Chapman L. Sargent-Cox K. Horswill M. S. Anstey K. J. (2014). The impact of age stereotypes on older adults' hazard perception performance and driving confidence. J. Appl. Gerontol. 35, 642–652. 10.1177/0733464813517505 24652925

[B13] Chrisler J. C. Barney A. Palatino B. (2016). Ageism can be hazardous to women's health: ageism, sexism, and stereotypes of older women in the healthcare system. J. Soc. Issues 72, 86–104. 10.1111/josi.12157

[B14] Cochran A. Hauschild T. Elder W. B. Neumayer L. A. Brasel K. J. Crandall M. L. (2013). Perceived gender-based barriers to careers in academic surgery. Am. J. Surg. 206, 263–268. 10.1016/j.amjsurg.2012.07.044 23414631

[B15] Coffman K. B. (2014). Evidence on self-stereotyping and the contribution of ideas. Q. J. Econ. 129, 1625–1660. 10.1093/qje/qju023

[B16] Cuddy A. J. Fiske S. T. (2002). Doddering but dear: process, content and function in stereotyping of older persons, in Ageism: Stereotyping and Prejudice Against Older Persons, ed T. Nelson (Cambridge, MA: MIT Press), 3–26.

[B17] Diekman A. B. Hirnisey L. (2007). The effect of context on the silver ceiling: a role congruity perspective on prejudiced responses. Pers. Soc. Psychol. Bull. 33, 1353–1366. 10.1177/0146167207303019 17933733

[B18] Doz Y. (2011). Qualitative research for international business. J. Int. Bus. Stud. 42, 582–590. 10.1057/jibs.2011.18

[B19] Dries N. Pepermans R. (2012). How to identify leadership potential: development and testing of a consensus model. Hum. Resour. Manage. 51, 361–385. 10.1002/hrm.21473

[B20] Duncan C. Loretto W. (2004). Never the right age? Gender and age-based discrimination in employment. Gender Work Organ. 11, 95–115. 10.1111/j.1468-0432.2004.00222.x

[B21] Eagly A. H. (2007). Female leadership advantage and disadvantage: resolving the contradictions. Psychol. Women Q. 31, 1–12. 10.1111/j.1471-6402.2007.00326.x

[B22] Eagly A. H. Karau S. J. (2002). Role congruity theory of prejudice toward female leaders. Psychol. Rev. 109, 573–598. 10.1037/0033-295X.109.3.573 12088246

[B23] Eagly A. H. Karau S. J. Makhijani M. G. (1995). Gender and the effectiveness of leaders: a meta-analysis. Psychol. Bull. 117, 125–145. 10.1037/0033-2909.117.1.125 7870858

[B24] Eagly A. H. Makhijani M. G. Klonsky B. G. (1992). Gender and the evaluation of leaders: a meta-analysis. Psychol. Bull. 111, 3 10.1016/j.leaqua.2003.09.004 7870858

[B25] Eaton A. A. Visser P. S. Burns V. (2017). How gender-role salience influences attitude strength and persuasive message processing. Psychol. Women Q. 41, 223–239. 10.1177/0361684317696257

[B26] Eccles J. S. Adler T. F. Futterman R. Goff S. B. Kaczala C. M. Meece J. L. (1983). Expectancies, values, and academic behaviors, in Achievement and Achievement Motivation, ed J. T. Spence (San Francisco, CA: Freeman), 75–146.

[B27] Eiksson T. Smith N. Smith V. (2017). Gender Stereotyping and Self-stereotyping Attitudes: A Large Field Study of Managers. (IZA Discussion Paper No. 10932). Retrieved from: https://www.iza.org/publications/dp/10932 (accessed March 22, 2019).

[B28] Elesser K. M. Lever J. (2011). Does gender bias against female leaders persist? Quantitative and qualitative data from a large-scale survey. Hum. Relat. 64, 1555–1578. 10.1177/0018726711424323

[B29] Emile M. Chalabaev A. Stephan Y. Corrion K. d'Arripe-Longueville F. (2014). Aging stereotypes and active lifestyle: personal correlates of stereotype internalization and relationships with level of physical activity among older adults. Psychol. Sport Exerc. 15, 198–204. 10.1016/j.psychsport.2013.11.002

[B30] Endendijk J. J. Groeneveld M. G. van Berkel S. R. Hallers-Haalboom E. T. Mesman J. Bakermans-Kranenburg M. J. (2013). Gender stereotypes in the family context: mothers, fathers, and siblings. Sex Roles 68, 577–590. 10.1007/s11199-013-0265-4

[B31] Finkelstein L. M. Burke M. J. Raju N. S. (1995). Age discrimination in simulated employment contexts: an integrative analysis. J. Appl. Psychol. 80, 652–663. 10.1037/0021-9010.80.6.652

[B32] Fiske S. T. Cuddy A. J. C. Glick P. Xu J. (2002). A model of (often mixed) stereotype content: competence and warmth respectively follow from perceived status and competition. J. Pers. Soc. Psychol. 82, 878–902. 10.1037/0022-3514.82.6.878 12051578

[B33] Fiske S. T. Xu J. Cuddy A. C. Glick P. (1999). (Dis) respecting versus (dis) liking: Status and interdependence predict ambivalent stereotypes of competence and warmth. J. Soc. Issues 55, 473–489. 10.1111/0022-4537.00128

[B34] Ganong L. Coleman M. (2005). Measuring intergenerational obligations. J. Marriage Fam. 67, 1003–1011. 10.1111/j.1741-3737.2005.00190.x

[B35] Ganong L. Coleman M. (2006). Multiple segment factorial vignette designs. J. Marriage Fam. 68, 455–468. 10.1111/j.1741-3737.2006.00264.x

[B36] Garstka T. A. Schmitt M. T. Branscombe N. R. Hummert M. L. (2004). How young and older adults differ in their responses to perceived age discrimination. Psychol. Aging 19, 326. 10.1037/0882-7974.19.2.326 15222826

[B37] Gaucher D. Friesen J. Kay A. C. (2011). Evidence that gendered wording in job advertisements exists and sustains gender inequality. J. Pers. Soc. Psychol. 101, 109–128. 10.1037/a0022530 21381851

[B38] Ghavami N. Peplau L. A. (2013). An intersectional analysis of gender and ethnic stereotypes: testing three hypotheses. Psychol. Women Q. 37(1), 113–127. 10.1177/0361684312464203

[B39] Gipson A. N. Pfaff D. L. Mendelsohn D. B. Catenacci L. T. Burke W. W. (2017). Women and leadership: selection, development, leadership style, and performance. J. Appl. Behav. Sci. 53, 32–65. 10.1177/0021886316687247

[B40] Gordon R. A. Arvey R. D. (2004). Age bias in laboratory and field settings: a meta-analytic investigation 1. J. Appl. Soc. Psychol. 34, 468–492. 10.1111/j.1559-1816.2004.tb02557.x

[B41] Gündemir S. Homan A. C. de Dreu C. K. W. van Vugt M. (2014). Think leader, think white? Capturing and weakening an implicit pro-white leadership bias. PLoS ONE 9:e83915. 10.1371/journal.pone.0083915 24416181 PMC3885528

[B42] Harnois C. E. (2014). Are perceptions of discrimination unidimensional, oppositional, or intersectional? Examining the relationship among perceived racial–ethnic-, gender-, and age-based discrimination. Sociol. Perspect. 57, 470–487. 10.1177/0731121414543028

[B43] Hayes A. F. (2013). Introduction to Mediation, Moderation, and Conditional Process Analysis: A Regression-Based Approach. New York, NY: The Guildford Press.

[B44] Heslin P. A. (2009). Potential” in the eye of the beholder: the role of managers who spot rising stars. Ind. Organ. Psychol. 2, 420–424. 10.1111/j.1754-9434.2009.01166.x

[B45] Hirschfeld R. R. Jordan M. H. Thomas C. H. Feild H. S. (2008). Observed leadership potential of personnel in a team setting: big five traits and proximal factors as predictors. Int. J. Select. Assess. 16, 385–402. 10.1111/j.1468-2389.2008.00443.x

[B46] Hirschfeld R. R. Thomas C. H. (2011). Age- and gender-based role incongruence: implications for knowledge mastery and observed leadership potential among personnel in a leadership development program. Pers. Psychol. 64, 661–692. 10.1111/j.1744-6570.2011.01222.x

[B47] Hoyt C. (2010). Women and leadership, in Leadership: Theory and Practice, 5th Edn, ed P. Northouse's (Thousand Oaks, CA: Sage), 301–333.

[B48] Kray L. Shirako A. (2011). Stereotype threat in organisations: an examination of its scope, triggers, and possible interventions, in Stereotype Threat: Theory, Process, and Application, ed M. Inzlicht and T. Schmader (New York, NY: Oxford University Press), 173–187.

[B49] Krings F. Sczesny S. Kluge A. (2011). Stereotypical inferences as mediators of age discrimination: the role of competence and warmth. Br. J. Manag. 22, 187–201. 10.1111/j.1467-8551.2010.00721.x

[B50] Kulik C. T. Perera S. Cregan C. (2016). Engage me: the mature-age worker and stereotype threat. Acad. Manag. J. 59, 2132–2156. 10.5465/amj.2015.0564

[B51] Kurtz-Costes B. Rowley S. J. Harris-Britt A. Woods T. A. (2008). Gender stereotypes about mathematics and science and self-perceptions of ability in late childhood and early adolescence. Merrill Palmer Q. 54, 386–409. 10.1353/mpq.0.0001

[B52] Levy B. (2009). Stereotype embodiment: a psychosocial approach to aging. Curr. Dir. Psychol. Sci. 18, 332–336. 10.1111/j.1467-8721.2009.01662.x 20802838 PMC2927354

[B53] Marcus J. Fritzsche B. A. Le H. Reeves M. D. (2016). Validation of the work-related age-based stereotypes (WAS) scale. J. Manager. Psychol. 31, 989–1004. 10.1108/JMP-11-2014-0320

[B54] Marques S. Lima M. L. Abrams D. Swift H. J. (2014). Aging stereotypes and will-to-live: effects over time. J. Appl. Soc. Psychol. 44, 399–408. 10.1111/jasp.12231

[B55] Martin A. E. North M. S. Phillips K. W. (2018). Intersectional escape: older women elude agentic prescriptions more than older men. Pers. Soc. Psychol. Bull. 45, 342–359. 10.1177/0146167218784895 30084290

[B56] Mast M. S. (2005). The world according to men: it is hierarchical and stereotypical. Sex Roles 53, 919–924. 10.1007/s11199-005-8310-6

[B57] McCann R Giles H (2002). Ageism in the workplace: a communication perspective, in Ageism: Stereotyping and Prejudice Against Older Persons, eds Nelson T. (Cambridge, MA: MIT Press), 163–199.

[B58] McKinsey Company (2015). Diversity Matters. Retrieved from: https://www.mckinsey.com/~/media/mckinsey/business%20functions/organization/our%20insights/why%20diversity%20matters/diversity%20matters.ashx

[B59] Mirza H. S. (2003). All the women are white, all the blacks are men–but some of us are brave: mapping the consequences of invisibility for black and minority ethnic women in Britain, in Explaining Ethnic Differences: Changing Patterns of Disadvantage in Britain, 121–138.

[B60] Mueller J. S. Goncalo J. A. Kamdar D. (2010). Recognizing creative leadership: can creative idea expression negatively relate to perceptions of leadership potential? J. Exp. Soc. Psychol. 47, 494–498. 10.1016/j.jesp.2010.11.010

[B61] Perry E. L. Golom F. D. Catenacci L. Ingraham M. E. Covais E. M. Molina J. J. (2017). Talkin'‘bout your generation: the impact of applicant age and generation on hiring-related perceptions and outcomes. Work Aging Retire. 3, 186–199. 10.1093/workar/waw029

[B62] Peters K. Haslam A. (2018). Research: To Be a Good Leader, Start by Being a Good Follower. Harvard Business Review, 2-4. Available online at: https://hbr.org/2018/08/research-to-be-a-good-leader-start-by-being-a-good-follower (accessed March 22, 2019).

[B63] Player A. Randsley de Moura G. Leite A. Abrams D. Tresh F (in press). Overlooked leadership potential: the preference for leadership potential in job candidates who are men vs. women. Front Psychol. 10.3389/fpsyg.2019.00755 PMC6476968 31040804

[B64] Polavieja J. G. (2012). Socially embedded investments: explaining gender differences in job-specific skills. Am. J. Sociol. 118, 592–634. 10.1086/667810

[B65] Posthuma R. A. Campion M. A. (2009). Age stereotypes in the workplace: common stereotypes, moderators, and future research directions. J. Manage. 35, 158–188. 10.1177/0149206308318617

[B66] Ridgeway C. L. (2011). Framed by Gender: How Gender Inequality Persists in the Modern World. New York, NY: Oxford University Press.

[B67] Robinson C. Fetters R. Riester D. Bracco A. (2009). The paradox of potential: a suggestion for guiding talent management discussions in organizations. Indus. Organ. Psychol. 2, 413–415. 10.1111/j.1754-9434.2009.01164.x

[B68] Salau O. Osibanjo A. Adeniji A. Olyudayo O. Faoloa H. Igbinoba E.. (2018). Data regarding talent management practices and innovation performance of academic staff in a technology-driven private university. Data Brief 19, 1040–1045. 10.1016/j.dib.2018.05.081 30225278 PMC6139485

[B69] Sarris A. Kirby N. (2005). Antarctica: a study of person-culture fit. Aust. J. Psychol. 57, 161–169. 10.1080/00049530500125165

[B70] Schein V. E. (1973). The relationship between sex role stereotypes and requisite management characteristics. J. Appl. Psychol. 57, 95–100. 10.1037/h0037128 4784761

[B71] Schein V. E. (1975). The relationship between sex role stereotypes and requisite management characteristics among female managers. J. Appl. Psychol. 60, 340–344. 10.1037/h0076637 1194167

[B72] Schwartz J. Collins L. Stockton H. Wagner D. Walsh B. (2017). Rewriting the Rules for the Digital Age: 2017 Deloitte Global Human Capital Trends. Retrieved from: https://www2.deloitte.com/am/en/pages/human-capital/articles/introduction-human-capital-trends-2017.html (accessed March 22, 2019).

[B73] Sczesny S. (2004). Gender stereotypes and the attribution of leadership traits: a cross-cultural comparison. Sex Roles 51, 631–645. 10.1007/s11199-004-0715-0

[B74] Silzer R. Church A. H. (2009). The potential for potential. Ind. Organ. Psychol. 2, 446–452. 10.1111/j.1754-9434.2009.01172.x

[B75] Simpson R. (2000). Gender mix and organizational fit: how gender imbalance at different levels of the organisation impacts on women managers. Women Manage. Rev. 15, 5–18. 10.1108/09649420010310173

[B76] Song J. Zuo B. Wen F. Yan L. (2017). Math-gender stereotypes and career intentions: an application of expectancy-value theory. Br. J. Guid. Counsel. 45, 328–340. 10.1080/03069885.2017.1283680

[B77] Spisak B. R. Grabo A. E. Arvey R. D. van Vugt M. (2014). The age of exploration and exploitation: younger-looking leaders endorsed for change and older-looking leaders endorsed for stability. Leadersh. Q. 25, 805–816. 10.1016/j.leaqua.2014.06.001

[B78] Stadler K. (2011). Talent reviews: the key to effective succession management. Bus. Strategy Ser. 12, 264–271. 10.1108/17515631111166906

[B79] Steele C. M. (1992). Race and the schooling of Black Americans. Atlantic Monthly 269, 68–78.

[B80] Steele C. M. (1997). A threat in the air: How stereotypes shape intellectual identity and performance. Am. Psychol. 52:613. 10.1037/0003-066X.52.6.613 9174398

[B81] Steffens N. K. Fonseca M. A. Ryan M. K. Rink F. A. Stoker J. I. Pieterse A. N. (2018). How feedback about leadership potential impacts ambition, organizational commitment, and performance. Leadersh. Q. 29, 637–647. 10.1016/j.leaqua.2018.06.001

[B82] Sun X. Xu D. Luo F. Wei Z. Wei C. Xue G. (2015). A cross-cultural perspective on the preference for potential effect: an individual participant data (IPD) meta-analysis approach. PLoS ONE 10:e0124170. 10.1371/journal.pone.0124170 25806521 PMC4373684

[B83] Swift H. Abrams D. Lamont R. Drury L. (2017). The risks of ageism model: how ageism and negative attitudes toward age can be a barrier to active aging. Soc. Issues Policy Rev. 11, 195–231. 10.1111/sipr.12031

[B84] Swift H. J. Abrams D. Marques S. (2013). Threat or boost? Social comparison affects older people's performance differently depending on task domain. J. Gerontol. B Psychol. Soc. Sci. 68, 23–30. 10.1093/geronb/gbs044 22512994

[B85] Tam T. (1997). Sex segregation and occupational gender inequality in the United States: devaluation or specialized training? Am. J. Sociol. 102, 1652–1692. 10.1086/231129

[B86] Thomas A. K. Smith A. M. Mazerolle M. (2018). The unexpected relationship between retrieval demands and memory performance when older adults are faced with age-related stereotypes. J. Gerontol. Psychol. Sci. 10.1093/geronb/gby031.. [Epub ahead of print]. 29608776

[B87] Tormala Z. L. Jia J. S. Norton M. I. (2012). The preference for potential. J. Pers. Soc. Psychol., 103, 567–583. 10.1037/a0029227 22775472

[B88] Tresh F. Randsley de Moura G. Leite C. Wyatt M. (2017). The intergroup dynamics of leadership potential. in Leadership: Bridging the Divide', EASP (Granada).

[B89] Tresh F. Randsley de Moura G. Leite C. Wyatt M. (2018). Perceptions of Leadership Potential: The Role of Sociodemographic Group Membership, Prototypicality and Performance. Manuscript in preparation.

[B90] Triguero-Sánchez R. Peña-Vinces J. Guillen J. (2018). How to improve firm performance through employee diversity and organizational culture. Rev. Business Manag. 20, 378–400. 10.7819/rbgn.v20i3.3303

[B91] Von Stockhausen L. Koeser S. Sczesny S. (2013). The gender typicality of faces and its impact on visual processing and on hiring decisions. Exp. Psychol. 60, 444–452. 10.1027/1618-3169/a000217 23895924

[B92] Warr P. Pennington J. (1993). Views about age discrimination and older workers, in Age and Employment: Policies, Attitudes, and Practice, eds Taylor P. Walker A. Casey B. Metcalf H. Lakey J. Warr P. Pennington J. (London: Institute of Personnel Management), 75–106.

[B93] World Economic Forum (2015). The Global Gender Gap Report 2015. Retrieved from World Economic Forum website: http://reports.weforum.org/global-gender-gap-report-2015/ (accessed March 22, 2019).

[B94] Wurm S. Tesch-Romer C. Tomasik M. J. (2007). Longitudinal findings on aging-related cognitions, control beliefs, and health in later life. J. Gerontol. 62, 156–164. 10.1093/geronb/62.3.P156 17507583

